# High species richness of tachinid parasitoids (Diptera: Calyptratae) sampled with a Malaise trap in Baihua Mountain Reserve, Beijing, China

**DOI:** 10.1038/s41598-021-01659-8

**Published:** 2021-11-12

**Authors:** Wenya Pei, Liping Yan, Thomas Pape, Qike Wang, Chuntian Zhang, Nan Yang, Fuxin Du, Dong Zhang

**Affiliations:** 1 School of Ecology and Nature Conservation, Beijing Forestry University, Beijing, China; 2 Natural History Museum of Denmark, University of Copenhagen, Copenhagen, Denmark; 3 School of BioSciences, University of Melbourne, Victoria, Australia; 4 College of Life Science, Shenyang Normal University, Shenyang, China; 5 Serving Officer in Administration Department of Baihua Mountain Reserve, Beijing, China

**Keywords:** Biodiversity, Ecology, Forest ecology

## Abstract

Tachinidae are one of the most speciose families of Diptera and the largest group of non-hymenopteran parasitoids. Little is known about their diversity, distribution patterns, and seasonal variation in most ecosystems. This study reports on tachinid flies collected by a Malaise trap over 73 weeks in Baihua Mountain Reserve, northern China, and investigates the patterns of local species richness and its temporal distribution. The most species-rich season was summer, but the majority of specimens were recovered in spring. A total of 755 tachinid specimens were collected, consisting of 144 species in 85 genera, comprising 26.5% of the species and 49.7% of the genera recorded from northern China. A total species richness of 243 was estimated, indicating that only a portion of the community of tachinid flies was collected at this location and suggesting that the diversity of tachinids might be underestimated across Beijing and northern China. This work is a first step in assessing patterns of tachinid diversity in China using quantitative sampling and establishes a baseline for comprehending the temporal and spatial diversity of these ecologically significant parasitoids.

## Introduction

More than 30 years ago, in a seminal paper, Edward O. Wilson^1^, a pioneer in biodiversity research, stated that insects were the little things that rule the world. Insects dominate terrestrial ecosystems in terms of numbers and species richness^2–5^. They have sparked wide interest because of their great diversity and importance in ecosystem function and stability, and the estimation of global insect species richness has been investigated for over 270 years^2,3,6–12^. Understanding the species richness and taxonomic composition of an ecosystem or in a region is key for its proper safeguarding and sustainable utilization^11–13^. Such studies can provide baseline data for local ecological processes and can be used to monitor the response of animal and plant communities to climate change, natural disturbances or intensification of anthropogenic activities^11,14^. Many researchers have demonstrated that species richness of insect communities changes over time, both seasonally and annually^15,16^. However, due to the logistic and financial challenges of long-term continuous sampling, few entomological surveys have been able to reveal the temporal complexity of insect communities^13,17–19^.

The methods of sampling are critically important for the accurate assessment of species richness of insect communities. Several methods have been widely applied in previous studies, including sweep nets, sticky traps, light traps, baited traps, pit-fall traps, Malaise traps, etc^20^. Among them, Malaise traps provide a relatively more systematic approach to surveying, and have been used successfully to monitor changes in the species composition of terrestrial communities and biodiversity assessments across the world^9,10^. Malaise traps are time- and cost-effective as they can continuously monitor an area with minimal human resources, as sampling efforts can be standardized by deploying them at fixed intervals. Thus, they are especially advantageous in the study of Diptera and Hymenoptera^21–25^. However, several studies on the effectiveness of Malaise traps have shown that less intensive trapping at a single site can provide sufficient information on the most abundant species^10,23^.

Tachinidae is an amazingly diverse Diptera family that contains exclusively parasitoid species and is distributed worldwide (ca. 8,500 described spp.)^26,27^. Tachinids are the largest group of non-hymenopteran parasitoids, and as parasitoids of herbivorous insects^28^, and thus are of great ecological importance in both natural and managed ecosystems. They primarily seek hosts among immature stages of herbivorous Lepidoptera, Hemiptera and Coleoptera^28–30^, and their diversity and population dynamics are suggested to reflect that of their hosts^30^. Understanding the temporal and spatial variation of tachinid diversity could indicate the health of local communities and may provide a basis for conservation work^31^. In addition, monitoring the temporal abundance of tachinids provides basic biological information on their voltinism, timing of development, and association with potential hosts, which is essential in assessing their effectiveness as potential means of biological control for pests^32^.

So far, only a few studies have quantified the species richness of local tachinids over different seasons in the Palaearctic Region^31–34^. In China, among the previous biodiversity surveys focused on tachinid species^35–41^, only a few documented or assessed the species richness and composition of tachinid communities in a certain geographic location. In this study, we collected tachinids using Malaise traps with continuous sampling over three seasons in the Baihua Mountain Reserve in Beijing, Northeastern China. Baihua Mountain Reserve forms an important ecological barrier for Beijing, and is known for its high biodiversity, environmental heterogeneity, and varied climate. This study aims to: (i) produce a species list as a baseline database of the Tachinidae in Baihua Mountain Reserve; (ii) estimate the total species richness of the tachinid community in the study area; (iii) examine how tachinid abundance and species richness vary over seasons; and (iv) assess voltinism and phenological patterns for several species of the tachinid community.

## Materials and methods

### Study area

**Figure 1:**
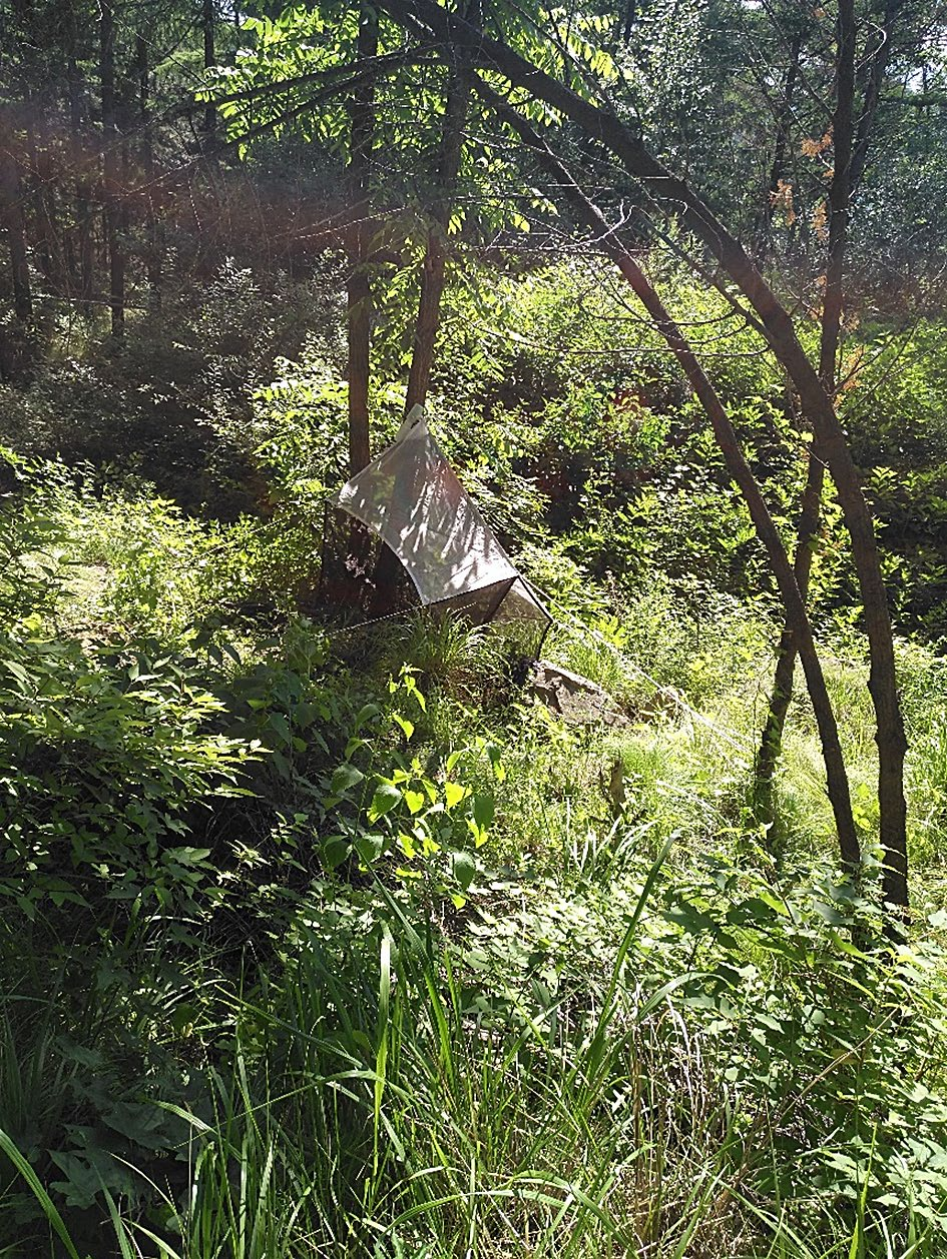
The Malaise trap deployed in the collecting site.

The sampling was conducted in Baihua Mountain Reserve, Northeastern China. The study site is located at the forest-farmland edge where grass and shrubland successions occurred after the artificial coniferous forests were cut down (N39°50′11.04″, E115°34′41.52″, 1224 m; Fig. 1). Common plant species in this study site are Rosaceae (*Spiraea* spp., *Rubus* spp., *Agrimonia* spp.), Saxifragaceae (*Deutzia* spp.), Dioscoreaceae (*Dioscorea* spp.), and Lamiaceae (*Phlomis* spp.). The large areas of woodland in the immediate vicinity of the site are dominated by trees of the families Pinaceae (*Larix* spp., *Pinus* spp.), Juglandaceae (*Juglans* spp.) and Salicaceae (*Populus* spp., *Salix* spp.).

### Specimen collecting

A single 1.8 m long and 1.7 m high Townes-type Malaise trap (Fig. 1) was deployed in the center of an open area approximately 20 m × 30 m for 73 weeks (4th June to 27th October in 2017 and from 6th July in 2018 to 6th July in 2019). Samples were collected every week and deposited in the Museum of Beijing Forestry University, Beijing, China. The field work permission was approved by the Baihua Mountain Reserve and was performed in accordance with relevant guidelines of the reserve.

### Identification

The systematic order and nomenclature follow those of Herting and Dely-Draskovits^42^ and O’Hara and Henderson^43^, and the genus level classification was based primarily on Tschorsnig and Richter^44^ and Cerretti et al.^45^ All the tachinid specimens were identified to the species level with integration of the recent literature^27,43,44,46–53^.

### Analysis

We used EstimateS 9.1.0 software package for Windows^54^ to estimate the total species richness based on the classic Chao 1 Richness Estimator (Chao-1), Incidence Coverage-based Estimator (ICE) and Abundance Coverage-based Estimator (ACE). The rarefaction curve and its 95% confidence intervals (95% CL) were calculated and plotted using 1,000 permutations. Each sample corresponded to the pooled catches from two weeks, thereby representing specimens collected the first and second half of each month respectively. To explore the patterns of species composition over time, we used a Non-metric Multidimensional Scaling (NMDS) ordination plot with the ‘vegan’^55^ package implemented in R, following construction of Bray–Curtis dissimilarity metrics. Differences in tachinid communities were further quantified by a Multi Response Permutation Procedure (MRPP) analysis^56^ using the ‘vegan’ package in R. MRPP was performed with 999 permutations on Bray–Curtis distance. The seasonal preference of different tachinid species was estimated by the indicator species analysis^57^ using PC-ORD (version 5.0) for Windows^58^. Only tachinid species with an observed indicator value (IV) > 50 and statistical significance P < 0.05 (in a Monte Carlo test (MCT) with 499 permutations) were considered to have a significant preference for a certain season.

## Results

### Species composition

**Figure 2:**
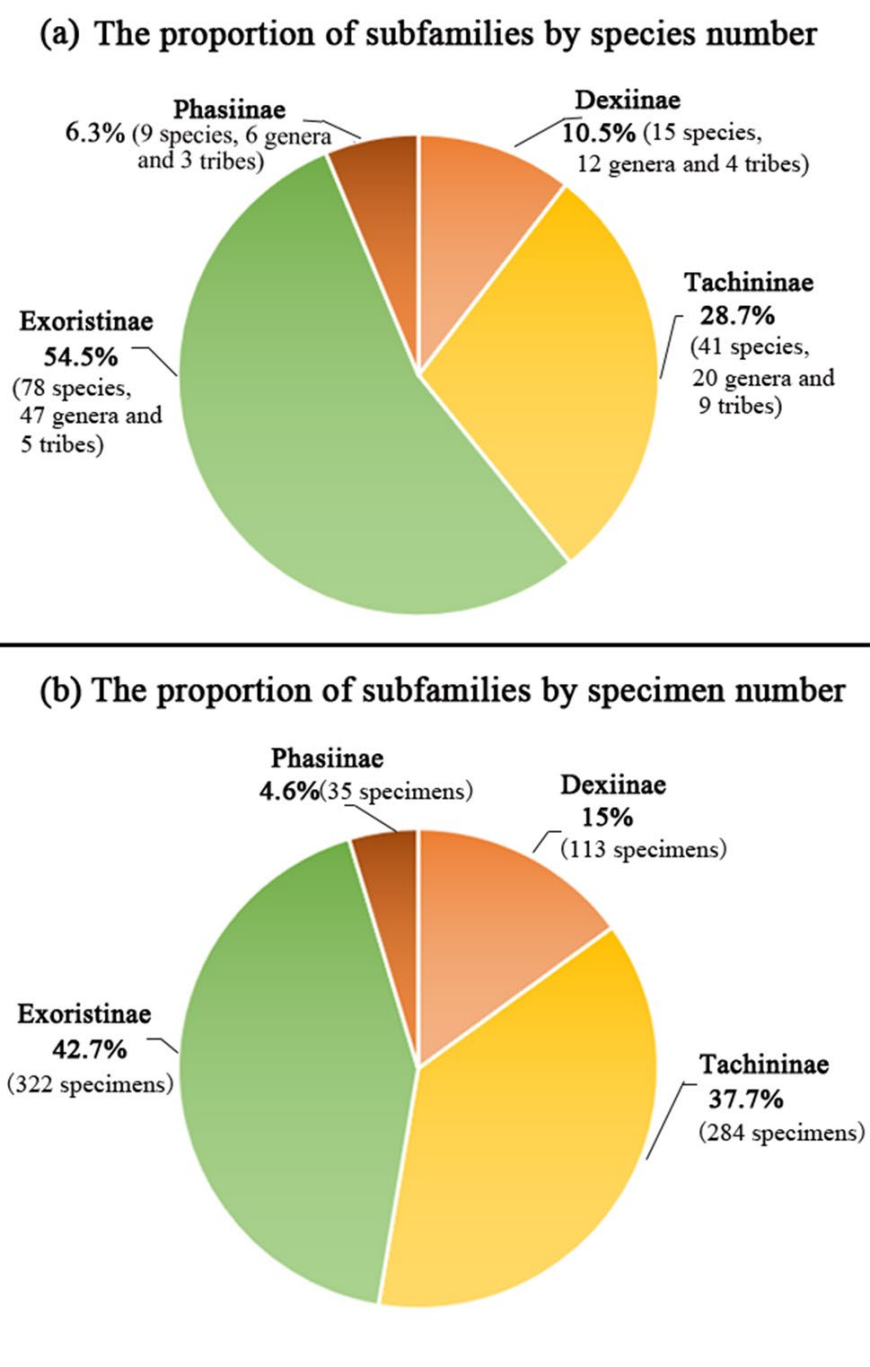
Pie chart of the proportion of each tachinid subfamily by (**a**) the number of species and (**b**) by the number of specimens.

**Figure 3:**
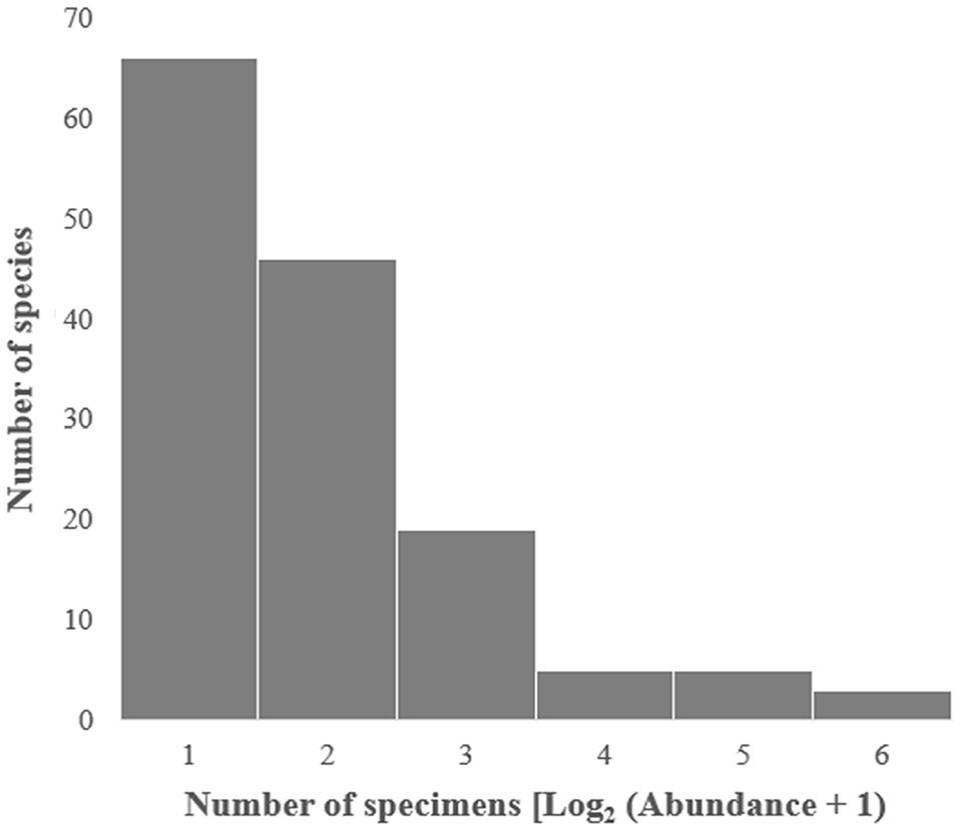
Rank-abundance of tachinid species sampled in this study. The x-axis is the log base two of the specimens abundance plus 1.

During the sampling period, no tachinid specimens were collected between 26th October 2018 and 28th February 2019. A total of 755 tachinid specimens were collected and sorted into 144 species (100 named species, 43 morphospecies, and one undescribed species) belonging to four subfamilies, 21 tribes and 85 genera (Supplementary Information File 1). We collected 248 specimens (73 spp.) in 2017 and 507 specimens (94 spp.) from July 2018 to July 2019 (Table 1). Exoristinae was the most abundant subfamily in terms of species richness (78 spp.) and abundance (322 specimens), followed by subfamily Tachininae (41 spp., 284 specimens) (Fig. 2). Subfamilies Dexiinae and Phasiinae comprised less than 20% of the material (24 spp., 148 specimens) (Fig. 2). At the tribal level, Blondeliini (Exoristinae) was the most speciose (31 spp.), followed by Goniini (Exoristinae) (24 spp.), while the most abundant were Siphonini (157 specimens) and Goniini (102 specimens). At the genus level, *Siphona* was both most speciose and most abundant (7 spp., 97 specimens). *Phorocera normalis* (Chao) was the most dominant species (89 specimens) during the sampling period (Supplementary Information File 1). Of the 144 species identified in this study, 45.8% were represented by only one specimen, 31.9% represented by two specimens, and only 11.8% were well represented by more than 10 specimens (Fig. 3).

**Table 1:** Estimates of total species richness of the tachinid community by specimens and samples for 2017, 2018, 2019, 2018–2019 and 2017–2019, based on 1,000 randomizations.

Estimator	2017		2018		2019		2018–2019		2017–2019	
	By specimens	By samples	By specimens	By samples	By specimens	By samples	By specimens	By samples	By specimens	By samples
No. of observations	248	10	188	8	319	8	507	16	755	26
Species observed	73	73	55	55	54	54	94	94	144	144
Chao-1	161	161	111	111	78	78	152	152	243	243
Chao-1 (lower–upper)	111–278	111–278	76–202	76–202	63–119	63–119	120–220	120–220	196–333	196–333

### Species richness

**Figure 4:**
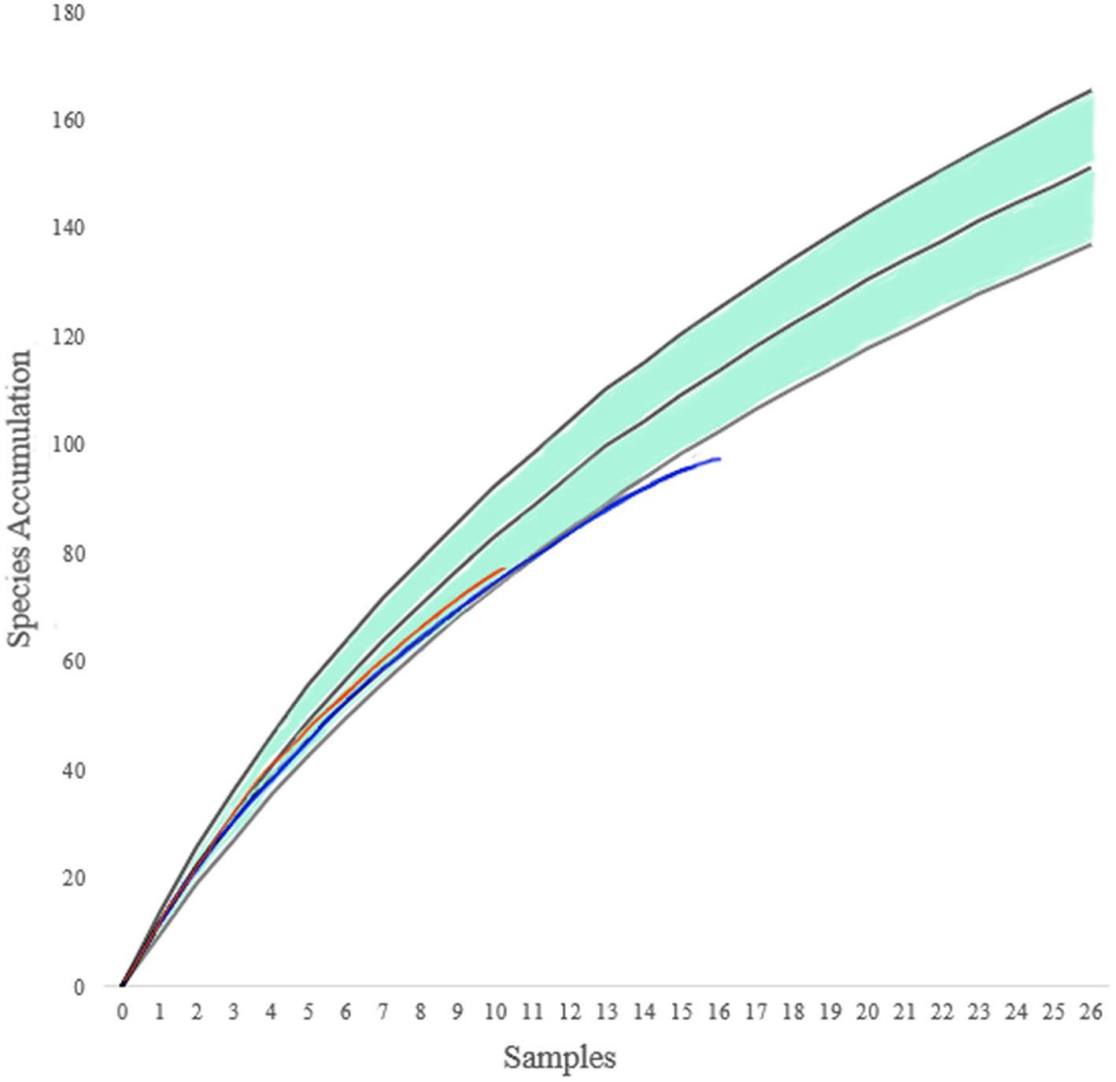
Accumulation curves of tachinid species against the number of samples. The green highlight is the species richness for 2017–2019 with the species richness accumulation in the center ± 95% CI. The red curve and the blue curve are the species richness accumulation curves for 2017 and 2018–2019, respectively. Curves are based on 1,000 randomizations.

**Figure 5:**
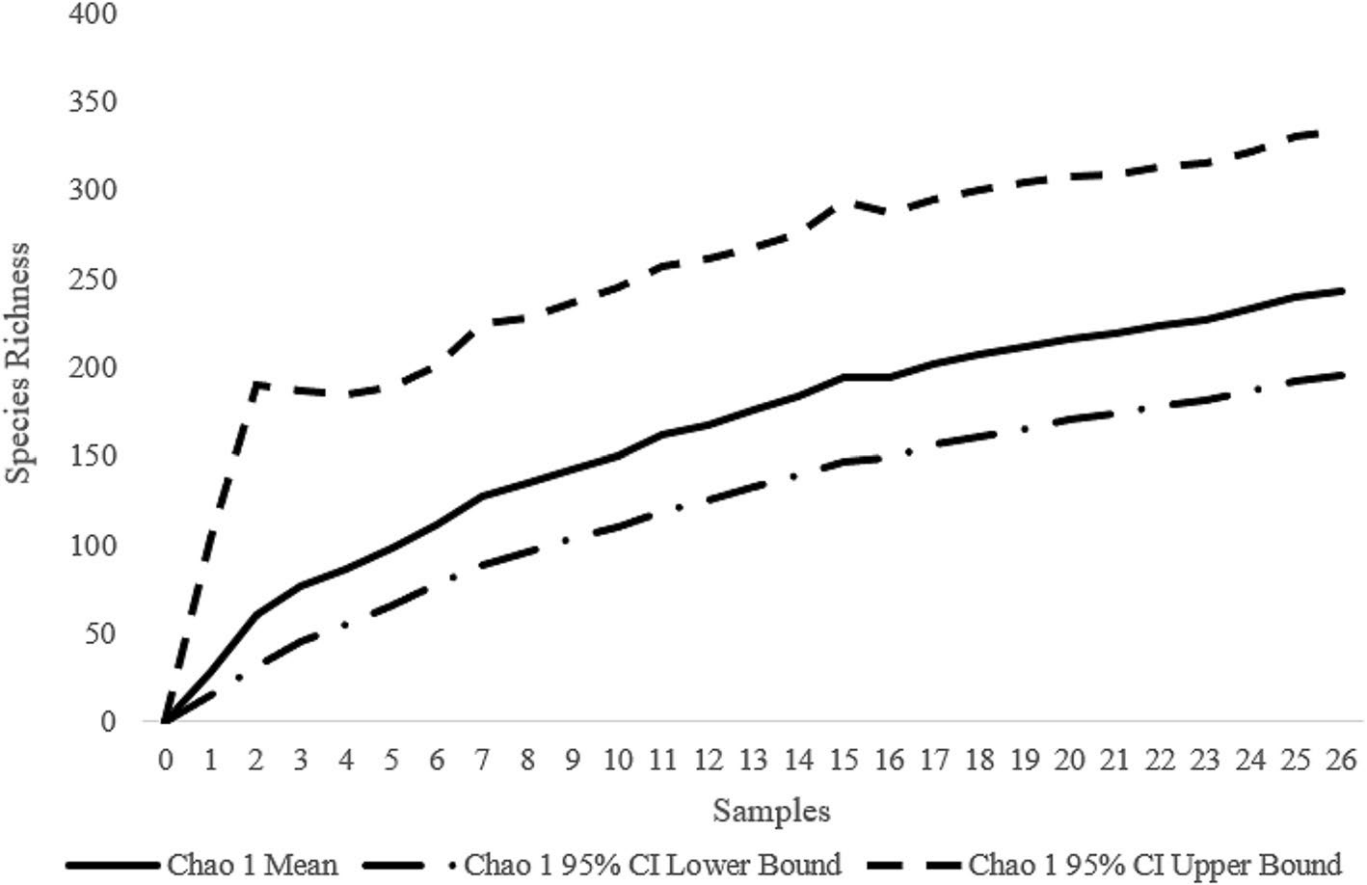
Estimated total species richness (Chao-1 estimator) with 95% confidence intervals for trap.

The species accumulation curve based on all samples does not near its asymptote (Fig. 4), indicating that only a portion of the species were collected. The total species richness at the sampling site, estimated using the sample-based Chao-1 estimator, is 243 species (95% CI 196–333; Fig. 5; Table 1). The results were similar using the ACE estimator (240 species) and the ICE estimator (257 species). The total species richness of the tachinid community in the area, estimated using the sample-based Chao-1 estimator, for the period from June 2017 to October 2017 was 161 species (95% CI 111–278; Fig. 5, Table 1), and from 6 July 2018 to 6 July 2019 was estimated to be 152 species (95% CI 120–220; Fig. 5, Table 1).

### Temporal distribution

Data analyses were based on all specimens captured between 2017 and 2019 (Supplementary Information File 1). Summer is the most species-rich season, with 257 species captured in the summer of 2017 (Table 2). Spring, conversely, is the season with the most specimens, with 257 specimens collected in the spring of 2019 (Table 2). Two Exoristinae species comprised almost 50% of all specimens collected in the spring of 2019; other dominant species were *Phorocera normalis* (31.1%) and *Phryno vetula* (16%).

**Table 2:** The number of tachinid species and specimens over summer 2017 to spring 2019.

Year	2017		2018	2019
Season	Summer	Fall	Fall	Spring
No. of species	62	21	38	27
No. of specimens	191	61	126	257

The data for the summer of 2018 and 2019 is insufficient, so there is no analysis carried out.

**Figure 6:**
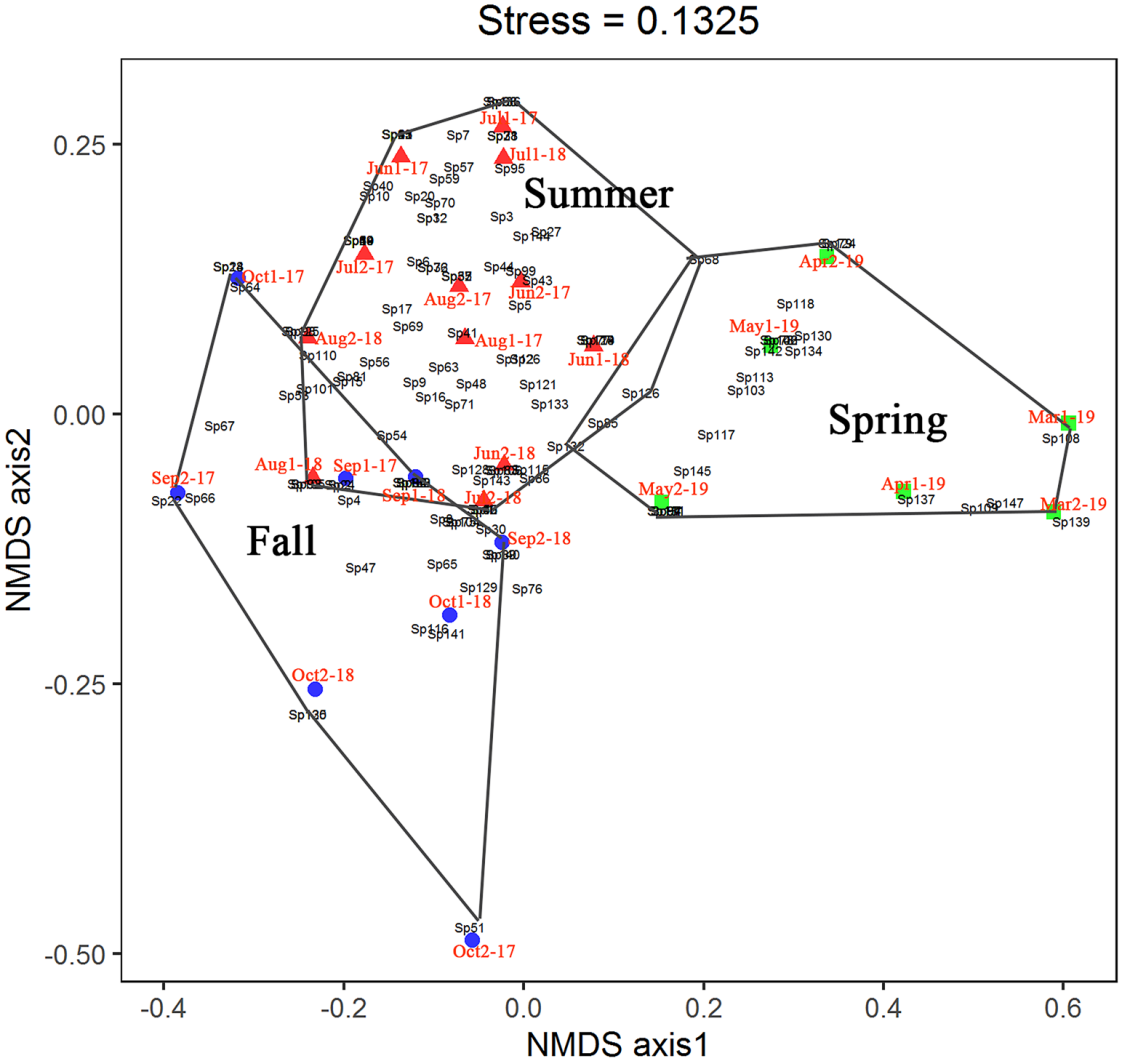
The 2-dimensional NMDS ordination configuration plot showing sample dates and points (black) for species using the Bray–Curtis dissimilarity matrix. Polygons group spring, summer and fall seasons.

The NMDS ordination for tachinid communities was plotted in Fig. 6. A clear division of the tachinid fauna were observed during the three seasons (Fig. 6). The stress obtained for this dimensionality was 0.1325, and the MRPP of the seasonal patterns observed in the NMDS plot was statistically significant (P = 0.001, A = 0.08723), indicating that the composition of the tachinid community varies significantly over seasons. Only 13 species were shared between spring and summer, eight species between spring and fall, 22 species between summer and fall, and only four species were shared among all three seasons.

The indicator species analysis was adopted to reveal the seasonal variation of dominant tachinid species. Three species dominated in spring: *Phorocera normalis* (IV = 100, MCT, P = 0.001), *Triarthria* sp.1 (IV = 65.3, MCT, P = 0.001) and *Panzeria mira* (IV = 64.9, MCT, P = 0.001). *Siphona* (*Siphona*) sp.2 is the unique dominant species in summer (IV = 52.2, MCT, P = 0.024) and *Prosena siberita* is the unique dominant species in fall (IV = 56.3, MCT, P = 0.008).

Based on the long-term research, we assessed voltinism and phenological patterns of several species of the tachinid community. *Admontia blanda*, *Admontia continuans*, *Carcelia bombylans*, *Ectophasia rotundiventris*, *Peribaea glabra* and *Siphona paludosa* have more than two generations per year (Supplementary Information File 1). *Ectophasia crassipennis*, *Leiophora innoxia*, *Pales carbonata* and *Pales pavida* may have two generations per year (Supplementary Information File 1). The species *Admontia gracilipes*, *Dinera xuei*, *Estheria magna*, *Hamaxiella brunnescens*, *Linnaemya picta*, *Panzeria mira*, *Pexopsis pollinis*, *Phebellia carceliaeformis*, *Phorocera normalis*, *Phryno vetula*, *Phytomyptera zonella*, *Prosena siberita*, *Tachina ursina*, and *Uromedina atrata* are likely to be univoltine (Supplementary Information File 1). In addition, we found that many species only appear in certain seasons. For example, *Phorocera normalis*, *Pexopsis pollinis*, *Tachina ursina*, *Gonia ussuriensis* were collected only in spring (Supplementary Information File 1). *Dinera xuei*, *Leiophora innoxia*, *Uromedina atrata*, *Linnaemya picta* were collected only in summer (Supplementary Information File 1). *Bithia modesta*, *Phytomyptera zonella*, *Peribaea tibialis* were collected only in fall (Supplementary Information File 1). Several of the potential bivoltine species were also seasonally restricted: *Pales pavida* exhibited split distributions over spring and fall, *Prosena siberita*, *Admontia blanda*, *Admontia continuans*, *Leiophora innoxia*, *Ectophasia crassipennis*, *Ectophasia rotundiventris*, *Siphona* (*Siphona*) *paludosa* were absent in spring, *Phryno vetula* and *Panzeria mira* were absent from the fall samples (Supplementary Information File 1). *Phorocera normalis* was the earliest species of the year that appeared, followed by *Tachina ursina* which appeared from 15 to 22 March 2019. *Ectophasia rotundiventris*, *Pales pavida*, *Graphogaster buccata*, *Phytomyptera zonella*, and *Suensonomyia nudinerva* were found in late fall (Supplementary Information File 1).

## Discussion

This study demonstrates the species richness and temporal distribution of Tachinidae sampled using a Malaise trap in Baihua Mountain Reserve over a sampling period of 73 weeks. A total of 755 tachinid individuals were collected and sorted into 144 species in 85 genera, including several species and genera newly recorded in China and one new species (Supplementary Information File 1 and Supplementary Information File 2). Our results indicate the effectiveness of Malaise traps in comprehensive insect community monitoring, and demonstrated that the Chinese tachinid fauna needs further study as more species are likely to be discovered.

The high species richness found in our study suggests that the species richness of tachinids in Beijing and northern China might be vastly underestimated. Initially, the number of species captured in this study is surprisingly high in comparison to the current local species records. The captured species number accounts for 55.8% and 81.7% of the published tachinid species and genera number in Beijing, 26.5% and 49.7% in northern China, and 11.5% and 30.4% of the tachinid species and genera number in China^59^. Such high percentages resulted from our sampling in a generally well-collected region, suggesting a largely undiscovered diversity of the tachinid species in this area. Another line of evidence is that the Chao-1 estimator indicated that the total tachinid species richness (Fig. 5, Table 1) in this single site is close to the current number of tachinid species recorded from across Beijing^59^. In fact, up to 77.8% of the species in our collection are represented by only one or two specimens (Fig. 3). The heavily skewed species abundance distribution curves (Figs. 3 and 4) suggest that many species in the community have not yet been sampled, and that the number of tachinid species will grow as the collecting time increases (Fig. 4).

The high number of species of Tachinidae in our study site might be correlated with the wide variety of climates and habitats located in Baihua Mountain Reserve^11,60^. This reserve contains various microclimates and microhabitats, including coniferous forest, open woodland, broad-leafed forest, grassland, and streams, with elevations ranging from 500 to 2043 m^61^. Our sampling site was located at the forest and farmland edge, with an abundance of wild open grassland that is important in maintaining a high level of insect biodiversity^62^. Although this edge habitat could encourage greater species richness and exotic species as it attracts insects from both open and forest-associated communities^63–66^, this location only represents two of the common habitats of this reserve. Therefore, the estimated 243 species from one single trapping site are very likely to be a subset of species occurring in the Baihua Mountain Reserve. The hyper-diversity of tachinid communities reinforces the needs for expanding monitoring efforts to uncover the diversity of not only tachinids, but also many other taxa in the region.

This study demonstrated the high sampling efficiency of Malaise traps. We recorded a relatively higher number of species in comparison to previous studies conducted in locations of similar latitudes over similar sampling periods^32,34,67,68^ (Table 3). For example, Stireman^34^ documented 79 species, and estimated a total of 122 species. Inclán and Stireman^32^ recorded a community of 117 species and estimated the total species to be 190. Despite the differences in the habitats (natural versus disrupted) between the previous studies^32,34^ and the current study, one explanation for this discrepancy may be the different sampling methods (pan traps versus Malaise traps). The pan trap is designed to catch insects which rely on visual orientation and active near the ground, while Malaise traps passively intercept insects flying at various heights^69–71^. Thus, the sampling efficiency of Malaise taps are higher than pan traps for Tachinidae biodiversity surveys.

**Table 3:** Summary information of tachinid species richness estimates from different zoogeographical regions.

Locality	Latitude	Elevation	Estimated species richness (Chao 1 or Chao 2 95% CI)			Diversity metrics		References
			Mean	Lower	Upper	eH’*	Simpson*	
Central Italy (understorey)	45°	26	52	–	150	–	–	Stireman et al.^67^
Ohio	39°	250	205	192	217	–	–	Inclán & Stireman^32^
Beijing	39°	1200	243	196	333	54.68	26.6	This study
Arizona	32°	1575	122.7	97	189	–	–	Stireman^34^
Maryland	39°	111.89	172.5	129.4	266.7	33.2	18.3	Burington et al.^68^
Ohio-1	39°	251	152.8	133.6	194.2	44.1	21	
Ohio-2	39°	269	116.6	92.9	173.2	23.7	9.9	
Arizona	32°	1575	135.0	102.0	215.4	32.3	18.2	
Costa Rica	10°	1600	440.1	386.6	523.3	133.7	57.1	
Ecuador-1	0°	2100	398.0	355.0	468.3	60.9	22.1	
Ecuador-2	0°	2173	240.0	193.0	327.2	95.6	68.2	
Ecuador-3	− 4°	3000	103.9	86.7	146.0	49.8	33.4	

The diversity and population dynamics of tachinids can reflect the hosts and their hostplants due to their trophic level as parasitoids of herbivorous insects^30^. Based on the most comprehensive study to date^52^, we found host information for only 50 of the 144 species recorded in this study (Supplementary Information File 4), comprising 810 host species belonging to six orders, 59 families and 450 genera (Supplementary Information File 4). Surprisingly, of the 50 species with known hosts, only 28 species had their host species also recorded in Baihua Mountain Reserve, suggesting the lack of host species studies of this remarkably diverse group of insects in Beijing and in north China. Understanding the relationship between these parasitoids and their hosts can be used as a baseline for the management of reserves, and as indicators of the diversity and population dynamics of their hosts^30^.

## Supplementary Information

Supplementary Information 1.

Supplementary Information 2.

Supplementary Information 3.

Supplementary Information 4.

## Data Availability

All data generated or analysed during this study are included in this published article (and its Supplementary Information files).
