# Supplementary material for: High species richness of tachinid parasitoids (Diptera: Calyptratae) sampled with a Malaise trap in Baihua Mountain Reserve, Beijing, China
Source: Sci Rep. 2021 Nov 12;11:22193. doi: 10.1038/s41598-021-01659-8 (PMC8590053; doi:10.1038/s41598-021-01659-8)
Supplement: Supplementary file 2 — Supplementary Information 2. [file 41598_2021_1659_MOESM2_ESM.docx]

Supporting Information

For

**High species richness of tachinid parasitoids (Diptera: Calyptratae) sampled with Malaise trap in Baihua Mountain Reserve, Beijing, China**

Content: Table A2

**Table A2** List of genera newly recorded for China and for Beijing.

| New to Beijing municipality | *Rondania* Robineau-Desvoidy, 1850 |
| --- | --- |
|  | *Cyrtophleba* Rondani, 1856 |
|  | *Admontia* Brauer *et* Bergenstamm, 1889 |
|  | *Biomeigenia* Mesnil, 1961 |
|  | *Dolichocoxys* Townsend, 1927 |
|  | *Leiophora* Robineau-Desvoidy, 1863 |
|  | *Opsomeigenia* Townsend, 1919 |
|  | *Phytorophaga* Bezzi, 1923 |
|  | *Trigonospila* Pokorny, 1886 |
|  | *Uromedina* Townsend, 1926 |
|  | *Gymnophryxe* Villeneuve, 1922 |
|  | *Weingaertneriella* Baranov, 1932 |
|  | *Phorocera* Robineau-Desvoidy, 1830 |
|  | *Catagonia* Brauer *et* Bergenstamm, 1891 |
|  | *Phryno* Robineau-Desvoidy, 1830 |
|  | *Suensonomyia* Mesnil, 1953 |
|  | *Subclytia* Pandellé, 1894 |
|  | *Graphogaster* Rondani, 1868 |
|  | *Bithia* Robineau-Desvoidy, 1863 |
|  | *Dolichopodomintho* Townsend, 1927 |
|  | *Sumpigaster* Macquart, 1855 |
|  | *Hamaxiella* Mesnil, 1967 |
|  | *Entomophaga* Lioy, 1864 |
| New to China | *Microsoma* Macquart, 1855 |
|  | *Gastrolepta* Rondani, 1862 |
|  | *Picconia* Robineau-Desvoidy, 1863 |
|  | *Ocytata* Gistel, 1848 |
|  | *Parapexopsis* Mesnil, 1953 |
|  | *Rhacodinella* Mesnil, 1968 |
|  | *Cistogaster* Latreille, 1829 |
|  | *Triarthria* Stephens, 1829 |
|  | *Mongolomintho* Richter, 1976 |
