# Supplementary material for: High species richness of tachinid parasitoids (Diptera: Calyptratae) sampled with a Malaise trap in Baihua Mountain Reserve, Beijing, China
Source: Sci Rep. 2021 Nov 12;11:22193. doi: 10.1038/s41598-021-01659-8 (PMC8590053; doi:10.1038/s41598-021-01659-8)
Supplement: Supplementary file 3 — Supplementary Information 3. [file 41598_2021_1659_MOESM3_ESM.docx]

Supporting Information

For

**High species richness of tachinid parasitoids (Diptera: Calyptratae) sampled with Malaise trap in Baihua Mountain Reserve, Beijing, China**

Content: Table A3

Table A3 (A) List of tribes were shared in two studies.

| Comparing Cerretti *et al*.^33^ and this study | Blondeliini |
| --- | --- |
|  | Eryciini |
|  | Goniini |
|  | Ernestiini |
|  | Macquartiini |
|  | Siphonini |
|  | Voriini |
|  | Cylindromyiini |
| Comparing Inclán & Stireman^32^ and this study | Blondeliini |
|  | Eryciini |
|  | Exoristini |
|  | Goniini |
|  | Winthemiini |
|  | Ernestiini |
|  | Graphogastrini |
|  | Leskiini |
|  | Minthoini |
|  | Siphonini |
|  | Tachinini |
|  | Dexiini |
|  | Voriini |
|  | Cylindromyiini |
|  | Phasiini |

| Comparing Cerretti *et al*.^33^ and this study | *Phorocera* Robineau-Desvoidy, 1830 |
| --- | --- |
|  | *Meigenia* Robineau-Desvoidy, 1830 |
|  | *Compsilura* Bouché, 1834 |
|  | *Vibrissina* Rondani, 1861 |
|  | *Nilea* Robineau-Desvoidy, 1863 |
|  | *Carcelia* Robineau-Desvoidy, 1830 |
|  | *Pales* Robineau-Desvoidy, 1830 |
|  | *Linnaemya* Robineau-Desvoidy, 1830 |
|  | *Macquartia* Robineau-Desvoidy, 1830 |
|  | *Triarthria* Stephens, 1829 |
|  | *Ceromya* Robineau-Desvoidy, 1830 |
|  | *Actia* Robineau-Desvoidy, 1830 |
|  | *Peribaea* Robineau-Desvoidy, 1863 |
|  | *Siphona* Meigen, 1803 |
|  | *Campylocheta* Rondani, 1859 |
|  | *Cyrtophleba* Rondani, 1856 |
|  | *Phasia* Latreille, 1804 |
| Comparing Inclán & Stireman^32^ and this study | *Admontia* Brauer et Bergenstamm, 1889 |
|  | *Blondelia* Robineau-Desvoidy, 1830 |
|  | *Lixophaga* Townsend, 1908 |
|  | *Medina* Robineau-Desvoidy, 1830 |
|  | *Opsomeigenia* Townsend, 1919 |
|  | *Vibrissina* Rondani, 1861 |
|  | *Carcelia* Robineau-Desvoidy, 1830 |
|  | *Nilea* Robineau-Desvoidy, 1863 |
|  | *Phebellia* Robineau-Desvoidy, 1846 |
|  | *Exorista* Meigen, 1803 |
|  | *Gonia* Meigen, 1803 |
|  | *Winthemia* Robineau-Desvoidy, 1830 |
|  | *Linnaemya* Robineau-Desvoidy, 1830 |
|  | *Phytomyptera* Rondani, 1845 |
|  | *Actia* Robineau-Desvoidy, 1830 |
|  | *Siphona* Meigen, 1803 |
|  | *Hemyda* Robineau-Desvoidy, 1830 |
|  | *Phasia* Latreille, 1804 |
|  | *Campylocheta* Rondani, 1859 |
|  | *Periscepsia* Gistel, 1848 |
|  | *Voria* Robineau-Desvoidy, 1830 |

Table A3 (B) List of genera were shared in two studies.
