# Supplementary material for: High species richness of tachinid parasitoids (Diptera: Calyptratae) sampled with a Malaise trap in Baihua Mountain Reserve, Beijing, China
Source: Sci Rep. 2021 Nov 12;11:22193. doi: 10.1038/s41598-021-01659-8 (PMC8590053; doi:10.1038/s41598-021-01659-8)
Supplement: Supplementary file 4 — Supplementary Information 4. [file 41598_2021_1659_MOESM4_ESM.docx]

Supporting Information

For

**High species richness of tachinid parasitoids (Diptera: Calyptratae) sampled with Malaise trap in Baihua Mountain Reserve, Beijing, China**

Content: Table A4

**Table A4** Host records of 50 tachinid flies.

| Tachinid species | Host catalogue | | |
| --- | --- | --- | --- |
|  | Order | Family | Species |
| *Dexia ventralis* Aldrich, 1925 | Coleoptera | Scarabaeidae | *Miridiba koreana* Niijima & Kinoshita |
|  |  |  | *Popillia japonica* Newman |
|  |  |  | *Popillia quadriguttata* Fabricius |
| *Prosena siberita* (Fabricius, 1775) | Coleoptera | Scarabaeidae | *Anomala dubia* Scopoli |
|  |  |  | *Anomala geniculata* Motschulsky |
|  |  |  | *Anomala luculenta* Erichson |
|  |  |  | *Anomala rufocuprea* Motschulsky |
|  |  |  | *Brahmina agnella* Faldermann |
|  |  |  | *Lasiopsis henningi* Fischer |
|  |  |  | *Mimela holosericea* Fabricius |
|  |  |  | *Mimela testaceipes* Motschulsky |
|  |  |  | *Oryctes rhinoceros* Linnaeus |
|  |  |  | *Popillia japonica* Newman |
| *Redtenbacheria insignis* Egger, 1861 | Heteroptera | Acanthosomatidae | *Acanthosoma denticaudum* Jakovlev |
|  |  | Pentatomidae | *Lelia decempunctata* Motschulsky |
|  |  |  | *Menida disjecta* Uhler |
| *Voria ruralis* (Fallén, 1810) | Lepidoptera | Arctiidae | *Arctia caja* Linnaeus |
|  |  |  | *Arctia festiva* Hufnagel |
|  |  |  | *Diaphora mendica* Clerck |
|  |  | Noctuidae | *Abrostola tripartita* Hufnagel |
|  |  |  | *Agrochola* sp.1 |
|  |  |  | *Autographa gamma* Linnaeus |
|  |  |  | *Autographa jota* Linnaeus |
|  |  |  | *Autographa pulchrina* Haworth |
|  |  |  | *Chrysodeixis chalcites* Esper |
|  |  |  | *Ctenoplusia agnata* Staudinger |
|  |  |  | *Diachrysia chrysitis* Linnaeus |
|  |  |  | *Diachrysia chryson* Esper |
|  |  |  | *Euchalcia variabilis* Piller |
|  |  |  | *Hecatera dysodea* Denis & Schiffermüller |
|  |  |  | *Helicoverpa armigera* Hübner |
|  |  |  | *Lacanobia oleracea* Linnaeus |
|  |  |  | *Macdunnoughia confusa* Stephens |
|  |  |  | *Mamestra brassicae* Linnaeus |
|  |  |  | *Ochropleura plecta* Linnaeus |
|  |  |  | *Panchrysia aurea* Hübner |
|  |  |  | *Plusia festucae* Linnaeus |
|  |  |  | *Spodoptera exigua* Hübner |
|  |  |  | *Syngrapha interrogationis* Linnaeus |
|  |  |  | *Xestia c-nigrum* Linnaeus |
| *Admontia blanda* (Fallén, 1820) | Diptera | Tipulidae | *Nephrotoma pratensis* Linnaeus |
| *Admontia grandicornis* (Zetterstedt, 1849) | Diptera | Tipulidae | *Tipula nubeculosa* Meigen |
| *Admontia maculisquama* (Zetterstedt, 1859) | Diptera | Tipulidae | *Tipula irrorata* Macquart |
|  |  |  | *Tipula lunata* Linnaeus |
|  |  |  | *Tipula pseudovariipennis* Czizek |
| *Biomeigenia gynandromima* Mesnil, 1961 | Coleoptera | Scarabaeidae | *Holotrichia diomphalia* Bates |
| *Blondelia inclusa* (Hartig, 1838) | Hymenoptera | Diprionidae | *Diprion pini* Linnaeus |
|  |  |  | *Gilpinia frutetorum* Fabricius |
|  |  |  | *Gilpinia laricis* Jurine |
|  |  |  | *Gilpinia pallida* Klug |
|  |  |  | *Gilpinia polytoma* Hartig |
|  |  |  | *Gilpinia socia* Klug |
|  |  |  | *Gilpinia variegata* Hartig |
|  |  |  | *Gilpinia virens* Klug |
|  |  |  | *Microdiprion pallipes* Fallén |
|  |  |  | *Neodiprion sertifer* Geoffroy |
| *Compsilura concinnata* (Meigen, 1824) | Hymenoptera | Cimbicidae | *Cimbex femoratus* Linnaeus |
|  |  | Pamphiliidae | *Neurotoma saltuum* Linnaeus |
|  |  | Tenthredinidae | *Allantus cinctus* Linnaeus |
|  |  |  | *Amauronematus stenogaster* Förster |
|  |  |  | *Cladius grandis* Serville |
|  |  |  | *Cladius pallipes* Serville |
|  |  |  | *Craesus septentrionalis* Linnaeus |
|  |  |  | *Pachynematus* sp.1 |
|  |  |  | *Strongylogaster osmundae* Takeuchi |
|  | Lepidoptera | Arctiidae | *Arctia caja* Linnaeus |
|  |  |  | *Arctia festiva* Hufnagel |
|  |  |  | *Arctia villica* Linnaeus |
|  |  |  | *Callimorpha dominula* Linnaeus |
|  |  |  | *Diaphora mendica* Clerck |
|  |  |  | *Hyphantria cunea* Drury |
|  |  |  | *Lemyra imparilis* Butler |
|  |  |  | *Lemyra inaequalis* Butler |
|  |  |  | *Lithosia quadra* Linnaeus |
|  |  |  | *Spilosoma lubricipeda* Linnaeus |
|  |  |  | *Spilosoma luteum* Hufnagel |
|  |  |  | *Spilosoma virginica* Fabricius |
|  |  |  | *Tyria jacobaeae* Linnaeus |
|  |  |  | *Watsonarctia deserta* Bartel |
|  |  | Bombycidae | *Bombyx mori* Linnaeus |
|  |  | Crambidae | *Anania terrealis* Treitschke |
|  |  |  | *Cnaphalocrocis medinalis* Guenée |
|  |  |  | *Cydalima perspectalis* Walker |
|  |  |  | *Ostrinia furnacalis* Guenée |
|  |  | Danaidae | *Danaus chrysippus* Linnaeus |
|  |  |  | *Parantica sita* Kollar |
|  |  | Drepanidae | Drepanidae sp.1 |
|  |  | Gelechiidae | *Helcystogramma triannulella* Herrich-Schäffer |
|  |  | Geometridae | *Abraxas grossulariata* Linnaeus |
|  |  |  | *Agriopis leucophaearia* Denis & Schiffermüller |
|  |  |  | *Archiearis parthenias* Linnaeus |
|  |  |  | *Arichanna melanaria* Linnaeus |
|  |  |  | *Ascotis selenaria* Denis & Schiffermüller |
|  |  |  | *Cepphis advenaria* Hübner |
|  |  |  | *Colotois pennaria* Linnaeus |
|  |  |  | *Cyclophora punctaria* Linnaeus |
|  |  |  | *Cystidia truncangulata* Wehrli |
|  |  |  | *Ectropis crepuscularia* Denis & Schiffermüller |
|  |  |  | *Ematurga atomaria* Linnaeus |
|  |  |  | *Ennomos erosaria* Denis & Schiffermüller |
|  |  |  | *Erannis defoliaria* Clerck |
|  |  |  | *Hydria undulata* Linnaeus |
|  |  |  | *Lycia hirtaria* Clerck |
|  |  |  | *Macaria liturata* Clerck |
|  |  |  | *Macaria notata* Linnaeus |
|  |  |  | *Phigalia pilosaria* Denis & Schiffermüller |
|  |  |  | *Phigalia verecundaria* Leech |
|  |  |  | *Timandra griseata* Petersen |
|  |  |  | *Triphosa dubitata* Linnaeus |
|  |  | Hepialidae | *Hepialus humuli* Linnaeus |
|  |  | Hesperiidae | *Carcharodus alceae* Esper |
|  |  |  | *Carterocephalus silvicola* Meigen |
|  |  |  | *Parnara guttata* Bremer & Grey |
|  |  |  | *Thoressa varia* Murray |
|  |  | Heterogynidae | *Heterogynis canalensis* Chapman |
|  |  |  | *Heterogynis penella* Hübner |
|  |  | Lasiocampidae | *Dendrolimus pini* Linnaeus |
|  |  |  | *Dendrolimus punctatus* Walker |
|  |  |  | *Dendrolimus spectabilis* Butler |
|  |  |  | *Dendrolimus superans* Butler |
|  |  |  | *Dendrolimus tabulaeformis* Tsai & Liu |
|  |  |  | *Eriogaster lanestris* Linnaeus |
|  |  |  | *Gastropacha populifolia* Esper |
|  |  |  | *Gastropacha quercifolia* Linnaeus |
|  |  |  | *Lasiocampa quercus* Linnaeus |
|  |  |  | *Macrothylacia rubi* Linnaeus |
|  |  |  | *Malacosoma neustria* Linnaeus |
|  |  |  | *Poecilocampa populi* Linnaeus |
|  |  |  | *Trichiura crataegi* Linnaeus |
|  |  | Libytheidae | *Libythea celtis* Laicharting |
|  |  | Limacodidae | *Parasa consocia* Walker |
|  |  | Lycaenidae | *Callophrys avis* Chapman |
|  |  |  | *Favonius orientalis* Murray |
|  |  |  | *Neozephyrus japonicus* Murray |
|  |  |  | *Plebejus argyrognomon* Bergsträsser |
|  |  |  | *Ussuriana stygiana* Butler |
|  |  | Lymantriidae | *Calliteara pudibunda* Linnaeus |
|  |  |  | *Dicallomera fascelina* Linnaeus |
|  |  |  | *Euproctis chrysorrhoea* Linnaeus |
|  |  |  | *Euproctis similis* Fuessly |
|  |  |  | *Ivela auripes* Butler |
|  |  |  | *Leucoma candida* Staudinger |
|  |  |  | *Leucoma salicis* Linnaeus |
|  |  |  | *Leucoma wiltshirei* Collenette |
|  |  |  | *Lymantria dispar* Linnaeus |
|  |  |  | *Lymantria mathura* Moore |
|  |  |  | *Lymantria monacha* Linnaeus |
|  |  |  | *Ocnerogyia amanda* Staudinger |
|  |  |  | *Orgyia antiqua* Linnaeus |
|  |  |  | *Orgyia recens* Hübner |
|  |  |  | *Orgyia rupestris* Rambur |
|  |  |  | *Parocneria terebinthi* Freyer |
|  |  | Noctuidae | *Acronicta aceris* Linnaeus |
|  |  |  | *Acronicta adaucta* Warren |
|  |  |  | *Acronicta alni* Linnaeus |
|  |  |  | *Acronicta auricoma* Denis & Schiffermüller |
|  |  |  | *Acronicta cuspis* Hübner |
|  |  |  | *Acronicta euphorbiae* Denis & Schiffermüller |
|  |  |  | *Acronicta leporina* Linnaeus |
|  |  |  | *Acronicta megacephala* Denis & Schiffermüller |
|  |  |  | *Acronicta menyanthidis* Esper |
|  |  |  | *Acronicta psi* Linnaeus |
|  |  |  | *Acronicta rumicis* Linnaeus |
|  |  |  | *Acronicta tridens* Denis & Schiffermüller |
|  |  |  | *Amphipyra tragopoginis* Clerck |
|  |  |  | *Anarta myrtilli* Linnaeus |
|  |  |  | *Archanara dissoluta* Treitschke |
|  |  |  | *Asota ficus* Fabricius |
|  |  |  | *Asteroscopus sphinx* Hufnagel |
|  |  |  | *Autographa gamma* Linnaeus |
|  |  |  | *Autographa pulchrina* Haworth |
|  |  |  | *Calyptra gruesa* Draudt |
|  |  |  | *Calyptra lata* Butler |
|  |  |  | *Calyptra thalictri* Borkhausen |
|  |  |  | *Catocala promissa* Denis & Schiffermüller |
|  |  |  | *Ceramica pisi* Linnaeus |
|  |  |  | *Chrysodeixis chalcites* Esper |
|  |  |  | *Colocasia coryli* Linnaeus |
|  |  |  | *Cosmia trapezina* Linnaeus |
|  |  |  | *Craniophora ligustri* Denis & Schiffermüller |
|  |  |  | *Ctenoplusia albostriata* Bremer & Grey |
|  |  |  | *Cucullia argentea* Hufnagel |
|  |  |  | *Cucullia asteris* Denis & Schiffermüller |
|  |  |  | *Cucullia gnaphalii* Hübner |
|  |  |  | *Cucullia lactucae* Denis & Schiffermüller |
|  |  |  | *Cucullia lychnitis* Rambur |
|  |  |  | *Cucullia verbasci* Linnaeus |
|  |  |  | *Diloba caeruleocephala* Linnaeus |
|  |  |  | *Dipterygina cupreotincta* Sugi |
|  |  |  | *Dypterygia scabriuscula* Linnaeus |
|  |  |  | *Erythroplusia pyropia* Butler |
|  |  |  | *Eudocima tyrannus* Guenée |
|  |  |  | *Euplexia lucipara* Linnaeus |
|  |  |  | *Hadena bicruris* Hufnagel |
|  |  |  | *Hecatera dysodea* Denis & Schiffermüller |
|  |  |  | *Helicoverpa armigera* Hübner |
|  |  |  | *Herminia tarsicrinalis* Knoch |
|  |  |  | *Hydraecia micacea* Esper |
|  |  |  | *Hypena crassalis* Fabricius |
|  |  |  | *Hypena rostralis* Linnaeus |
|  |  |  | *Lacanobia oleracea* Linnaeus |
|  |  |  | *Mamestra brassicae* Linnaeus |
|  |  |  | *Melanchra persicariae* Linnaeus |
|  |  |  | *Mniotype adusta* Esper |
|  |  |  | *Moma alpium* Osbeck |
|  |  |  | *Mythimna separata* Walker |
|  |  |  | *Mythimna unipuncta* Haworth |
|  |  |  | *Noctua pronuba* Linnaeus |
|  |  |  | *Nonagria typhae* Thunberg |
|  |  |  | *Orthosia cerasi* Fabricius |
|  |  |  | *Orthosia cruda* Denis & Schiffermüller |
|  |  |  | *Orthosia gothica* Linnaeus |
|  |  |  | *Phlogophora meticulosa* Linnaeus |
|  |  |  | *Plusia festucae* Linnaeus |
|  |  |  | *Polymixis flavicincta* Denis & Schiffermüller |
|  |  |  | *Polypogon* sp.1 |
|  |  |  | *Pyrrhia bifasciata* Staudinger |
|  |  |  | *Scoliopteryx libatrix* Linnaeus |
|  |  |  | *Simyra albovenosa* Goeze |
|  |  |  | *Simyra nervosa* Denis & Schiffermüller |
|  |  |  | *Spodoptera littoralis* Boisduval |
|  |  |  | *Tiliacea citrago* Linnaeus |
|  |  |  | *Trachea atriplicis* Linnaeus |
|  |  |  | *Xestia c-nigrum* Linnaeus |
|  |  |  | *Xestia triangulum* Hufnagel |
|  |  |  | *Zanclognatha lunalis* Scopoli |
|  |  | Notodontidae | *Cerura vinula* Linnaeus |
|  |  |  | *Clostera anachoreta* Denis & Schiffermüller |
|  |  |  | *Drymonia ruficornis* Hufnagel |
|  |  |  | *Furcula bifida* Brahm |
|  |  |  | *Notodonta ziczac* Linnaeus |
|  |  |  | *Ochrostigma velitaris* Hufnagel |
|  |  |  | *Phalera bucephala* Linnaeus |
|  |  |  | *Phalera bucephaloides* Ochsenheimer |
|  |  |  | *Stauropus basalis* Moore |
|  |  |  | *Stauropus fagi* Linnaeus |
|  |  | Nymphalidae | *Aglais urticae* Linnaeus |
|  |  |  | *Apatura ilia D*enis & Schiffermüller |
|  |  |  | *Araschnia burejana* Bremer |
|  |  |  | *Araschnia levana* Linnaeus |
|  |  |  | *Argynnis hyperbius* Linnaeus |
|  |  |  | *Argynnis paphia* Linnaeus |
|  |  |  | *Charaxes jasius* Linnaeus |
|  |  |  | *Euphydryas aurinia* Rottemburg |
|  |  |  | *Euphydryas maturna* Linnaeus |
|  |  |  | *Inachis io* Linnaeus |
|  |  |  | *Kallima inachus* Boisduval |
|  |  |  | *Limenitis camilla* Linnaeus |
|  |  |  | *Limenitis reducta* Staudinger |
|  |  |  | *Melitaea athalia* Rottemburg |
|  |  |  | *Melitaea cinxia* Linnaeus |
|  |  |  | *Melitaea phoebe* Denis & Schiffermüller |
|  |  |  | *Neptis philyra* Ménétriés |
|  |  |  | *Neptis sappho* Pallas |
|  |  |  | *Nymphalis antiopa* Linnaeus |
|  |  |  | *Nymphalis canace* Linnaeus |
|  |  |  | *Nymphalis polychloros* Linnaeus |
|  |  |  | *Nymphalis xanthomelas* Esper |
|  |  |  | *Polygonia c*-*album* Linnaeus |
|  |  |  | *Vanessa atalanta* Linnaeus |
|  |  |  | *Vanessa cardui* Linnaeus |
|  |  |  | *Vanessa indica* Herbst |
|  |  | Papilionidae | *Iphiclides podalirius* Linnaeus |
|  |  |  | *Luehdorfia japonica* Leech |
|  |  |  | *Papilio alexanor* Esper |
|  |  |  | *Papilio machaon* Linnaeus |
|  |  |  | *Parnassius apollo* Linnaeus |
|  |  | Pieridae | *Aporia crataegi* Linnaeus |
|  |  |  | *Gonepteryx aspasia* Ménétriés |
|  |  |  | *Gonepteryx cleopatra* Linnaeus |
|  |  |  | *Gonepteryx rhamni* Linnaeus |
|  |  |  | *Pieris brassicae* Linnaeus |
|  |  |  | *Pieris Mannii* Mayer |
|  |  |  | *Pieris melete* Ménétriés |
|  |  |  | *Pieris napi* Linnaeus |
|  |  |  | *Pieris rapae* Linnaeus |
|  |  |  | *Pontia daplidice* Linnaeus |
|  |  | Psychidae | *Bambalina* sp.1 |
|  |  | Saturniidae | *Actias isabellae* Graells |
|  |  |  | *Samia cynthia* Drury |
|  |  |  | *Saturnia pavonia* Linnaeus |
|  |  |  | *Saturnia pyri* Denis & Schiffermüller |
|  |  | Satyridae | *Lasiommata maera* Linnaeus |
|  |  |  | *Lasiommata megera* Linnaeus |
|  |  |  | *Lethe diana* Butler |
|  |  |  | *Lethe marginalis* Motschulsky |
|  |  |  | *Melanitis leda* Linnaeus |
|  |  |  | *Melanitis phedima* Cramer |
|  |  |  | *Mycalesis gotama* Moore |
|  |  | Sphingidae | *Acherontia atropos* Linnaeus |
|  |  |  | *Clarina syriaca* Lederer |
|  |  |  | *Daphnis nerii* Linnaeus |
|  |  |  | *Deilephila elpenor* Linnaeus |
|  |  |  | *Hyles euphorbiae* Linnaeus |
|  |  |  | *Hyles galii* Rottemburg |
|  |  |  | *Laothoe populi* Linnaeus |
|  |  |  | *Macroglossum stellatarum* Linnaeus |
|  |  |  | *Mimas tiliae* Linnaeus |
|  |  |  | *Psilogramma menephron* Cramer |
|  |  |  | *Smerinthus planus* Walker |
|  |  |  | *Smerinthus ocellata* Linnaeus |
|  |  |  | *Sphinx ligustri* Linnaeus |
|  |  |  | *Sphinx pinastri* Linnaeus |
|  |  | Thaumetopoeidae | *Thaumetopoea bonjeani* Powell |
|  |  |  | *Thaumetopoea ispartaensis* Doğanlar & Avcl |
|  |  |  | *Thaumetopoea* Jordan*a* Staudinger |
|  |  |  | *Thaumetopoea pinivora* Treitschke |
|  |  |  | *Thaumetopoea pityocampa* Denis & Schiffermüller |
|  |  |  | *Thaumetopoea processionea* Linnaeus |
|  |  |  | *Thaumetopoea solitaria* Freyer |
|  |  |  | *Thaumetopoea wilkinsoni* Tams |
|  |  | Thyatiridae | *Polyploca ridens* Fabricius |
|  |  | Tortricidae | *Archips crataegana* Hübner |
|  |  |  | *Archips fuscocupreanus* Walsingham |
|  |  |  | *Archips oporana* Linnaeus |
|  |  |  | *Archips xylosteana* Linnaeus |
|  |  |  | *Choristoneura diversana* Hübner |
|  |  |  | *Choristoneura murinana* Hübner |
|  |  |  | *Tortrix viridana* Linnaeus |
|  |  | Yponomeutidae | *Yponomeuta cagnagella* Hübner |
|  |  |  | *Yponomeuta malinellus* Zeller |
|  |  |  | *Yponomeuta padella* Linnaeus |
|  |  |  | *Yponomeuta polystictus* Butler |
|  |  | Zygaenidae | *Aglaope infausta* Linnaeus |
|  |  |  | *Artona martini* Efetov |
|  |  |  | *Chalcosia remota* Walker |
|  |  |  | *Elcysma westwoodii* Vollenhoven |
|  |  |  | *Pryeria sinica* Moore |
|  |  |  | *Theresimima ampellophaga* Bayle-Barelle |
|  |  |  | *Zygaena carniolica* Scopoli |
|  |  |  | *Zygaena fausta* Linnaeus |
|  |  |  | *Zygaena fredi* Reiss |
|  |  |  | *Zygaena niphona* Butler |
|  |  |  | *Zygaena occitanica* Villers |
|  |  |  | *Zygaena purpuralis* Brünnich |
|  |  |  | *Zygaena viciae* Denis & Schiffermüller |
| *Gastrolepta anthracina* (Meigen, 1826) | Coleoptera | Tenebrionidae | *Lagria hirta* Linnaeus |
| *Istocheta nyctia* (Borisova-Zinov’eva, 1966) | Coleoptera | Scarabaeidae | *Brahmina crenicollis* Motschulsky |
| *Leiophora innoxia* (Meigen, 1824) | Orthoptera | Tetrigidae | *Tetrix bipunctata* Linnaeus |
|  |  |  | *Tetrix tenuicornis* Sahlberg |
|  |  |  | *Tetrix undulata* Sowerby |
| *Lixophaga latigena* Shima, 1979 | Coleoptera | Curculionidae | *Curculio sikkimensis* Heller |
| *Meigenia dorsalis* (Meigen, 1824) | Coleoptera | Chrysomelidae | *Cercyonops caraganae* Gebler |
|  |  |  | *Chrysolina americana* Linnaeus |
|  |  |  | *Chrysolina montana* Gebler |
|  |  |  | *Chrysolina varians* Schaller |
|  |  |  | *Chrysomela populi* Linnaeus |
|  |  |  | *Chrysomela salicivorax* Fairmaire |
|  |  |  | *Crioceris quatuordecimpunctata* Scopoli |
|  |  |  | *Entomoscelis orientalis* Motschulsky |
|  |  |  | *Gonioctena decemnotata* Marsham |
|  |  |  | *Gonioctena viminalis* Linnaeus |
|  |  |  | *Plagiosterna aenea* Linnaeus |
| *Meigenia mutabilis* (Fallén, 1810) | Coleoptera | Chrysomelidae | *Agelastica alni* Linnaeus |
|  |  |  | *Blepharida sacra* Weise |
|  |  |  | *Chrysolina americana* Linnaeus |
|  |  |  | *Chrysolina didymata* Scriba |
|  |  |  | *Chrysolina fastuosa* Scopoli |
|  |  |  | *Chrysolina herbacea* Duftschmid |
|  |  |  | *Chrysolina hyperici* Forster |
|  |  |  | *Chrysolina polita* Linnaeus |
|  |  |  | *Chrysolina varians* Schaller |
|  |  |  | *Chrysomela populi* Linnaeus |
|  |  |  | *Chrysomela saliceti* Weise |
|  |  |  | *Chrysomela tremulae* Fabricius |
|  |  |  | *Colaphus sophiae* Schaller |
|  |  |  | *Colaspidema barbarum* Fabricius |
|  |  |  | *Crioceris asparagi* Linnaeus |
|  |  |  | *Crioceris duodecimpunctata* Linnaeus |
|  |  |  | *Crioceris quatuordecimpunctata* Scopoli |
|  |  |  | *Entomoscelis adonidis* Pallas |
|  |  |  | *Gastrophysa polygoni* Linnaeus |
|  |  |  | *Gastrophysa viridula* De Geer |
|  |  |  | *Gonioctena decemnotata* Marsham |
|  |  |  | *Gonioctena fornicata* Brüggemann |
|  |  |  | *Gonioctena linnaeana* Schrank |
|  |  |  | *Gonioctena olivacea* Forster |
|  |  |  | *Gonioctena pallida* Linnaeus |
|  |  |  | *Gonioctena quinquepunctata* Fabricius |
|  |  |  | *Gonioctena viminalis* Linnaeus |
|  |  |  | *Hydrothassa marginella* Linnaeus |
|  |  |  | *Lilioceris merdigera* Linnaeus |
|  |  |  | *Oulema melanopus* Linnaeus |
|  |  |  | *Phaedon cochleariae* Fabricius |
|  |  |  | *Phaedon tumidulus* Germar |
|  |  |  | *Phratora vitellinae* Linnaeus |
|  |  |  | *Phratora vulgatissima* Linnaeus |
|  |  |  | *Plagiodera versicolora* Laicharting |
|  |  |  | *Plagiosterna aenea* Linnaeus |
|  |  |  | *Pyrrhalta viburni* Paykull |
|  |  |  | *Timarcha goettingensis* Linnaeus |
|  |  | Curculionidae | *Hypera postica* Gyllenhal |
|  |  |  | *Hypera rumicis* Linnaeus |
|  | Hymenoptera | Tenthredinidae | *Athalia cornubiae* Benson |
|  |  |  | *Athalia liberta* Klug |
|  |  |  | *Athalia rosae* Linnaeus |
| *Vibrissina turrita* (Meigen, 1824) | Hymenoptera | Argidae | *Arge enodis* Linnaeus |
|  |  |  | *Arge fuscipes* Fallén |
|  |  |  | *Arge nigripes* Retzius |
|  |  |  | *Arge ochropus* Gmelin |
|  |  |  | *Arge pagana* Panzer |
|  |  |  | *Arge pullata* Zaddach |
|  |  |  | *Arge shawi* Liston |
|  |  |  | *Arge ustulata* Linnaeus |
|  |  | Cimbicidae | *Cimbex taukushi* Marlatt |
|  |  | Tenthredinidae | *Allantus cinctus* Linnaeus |
|  |  |  | *Allantus luctifer* Smith |
|  |  |  | *Ametastegia equiseti* Fall*én* |
|  |  |  | *Aneugmenus* sp.1 |
|  |  |  | *Athalia cordata* Serville |
|  |  |  | *Athalia infumata* Marlatt |
|  |  |  | *Athalia japonica* Klug |
|  |  |  | *Athalia rosae* Linnaeus |
|  |  |  | *Caliroa annulipes* Klug |
|  |  |  | *Caliroa cerasi* Linnaeus |
|  |  |  | *Craesus alniastri* Scharfenberg |
|  |  |  | *Craesus japonicus* Takeuchi |
|  |  |  | *Craesus septentrionalis* Linnaeus |
|  |  |  | *Eriocampa ovata* Linnaeus |
|  |  |  | *Macrophya albicincta* Schrank |
|  |  |  | *Monophadnus spinolae* Klug |
|  |  |  | *Monostegia abdominalis* Fabricius |
|  |  |  | *Phymatocera aterrima* Klug |
|  |  |  | *Pristiphora erichsonii* Hartig |
|  |  |  | *Rhadinoceraea micans* Klug |
| *Carcelia bombylans* Robineau-Desvoidy, 1830 | Lepidoptera | Arctiidae | *Hyphantria cunea* Drury |
|  |  |  | *Lemyra inaequalis* Butler |
|  |  |  | *Phragmatobia fuliginosa* Linnaeus |
|  |  |  | *Spilosoma lubricipeda* Linnaeus |
|  |  |  | *Spilosoma luteum* Hufnagel |
|  |  | Lasiocampidae | *Malacosoma neustria* Linnaeus |
|  |  | Lymantriidae | *Euproctis pulverea* Leech |
|  |  |  | *Ivela auripes* Butler |
|  |  |  | *Orgyia thyellina* Butler |
| *Carcelia gnava* (Meigen, 1824) | Lepidoptera | Arctiidae | *Arctia caja* Linnaeus |
|  |  |  | *Hyphantria cunea* Drury |
|  |  |  | *Spilosoma lubricipeda* Linnaeus |
|  |  | Lasiocampidae | *Kunugia undans* Walker |
|  |  |  | *Malacosoma castrensis* Linnaeus |
|  |  |  | *Malacosoma neustria* Linnaeus |
|  |  | Lymantriidae | *Calliteara lunulata* Butler |
|  |  |  | *Calliteara pseudabietis* Butler |
|  |  |  | *Calliteara pudibunda* Linnaeus |
|  |  |  | *Euproctis subflava* Bremer |
|  |  |  | *Ivela auripes* Butler |
|  |  |  | *Leucoma salicis* Linnaeus |
|  |  |  | *Lymantria dispar* Linnaeus |
|  |  |  | *Lymantria fumida* Butler |
|  |  |  | *Lymantria mathura* Moore |
|  |  |  | *Orgyia antiqua* Linnaeus |
|  |  |  | *Orgyia antiquoides* Hübner |
|  |  |  | *Orgyia recens* Hübner |
|  |  | Noctuidae | *Cucullia* sp.1 |
|  |  |  | *Xestia c*-*nigrum* Linnaeus |
|  |  | Notodontidae | *Phalera bucephala* Linnaeus |
|  |  | Saturniidae | *Saturnia japonica* Moore |
|  |  | Thyatiridae | *Polyploca ridens* Fabricius |
| *Carcelia rasa* (Macquart, 1849) | Lepidoptera | Arctiidae | *Arctia caja* Linnaeus |
|  |  |  | *Lemyra imparilis* Butler |
|  |  |  | *Lemyra inaequalis* Butler |
|  |  |  | *Spilarctia obliqua* Walker |
|  |  |  | *Spilosoma* sp.1 |
|  |  | Lasiocampidae | *Eriogaster catax* Linnaeus |
|  |  |  | *Kunugia undans* Walker |
|  |  |  | *Malacosoma neustria* Linnaeus |
|  |  | Lymantriidae | *Calliteara argentata* Butler |
|  |  |  | *Calliteara lunulata* Butler |
|  |  |  | *Calliteara pseudabietis* Butler |
|  |  |  | *Calliteara pudibunda* Linnaeus |
|  |  |  | *Dicallomera fascelina* Linnaeus |
|  |  |  | *Euproctis chrysorrhoea* Linnaeus |
|  |  |  | *Euproctis similis* Fuessly |
|  |  |  | *Euproctis subflava* Bremer |
|  |  |  | *Leucoma ochropoda* Eversmann |
|  |  |  | *Laelia miyanoi* Kishida |
|  |  |  | *Lymantria dispar* Linnaeus |
|  |  |  | *Lymantria mathura* Moore |
|  |  |  | *Lymantria monacha* Linnaeus |
|  |  |  | *Orgyia antiqua* Linnaeus |
|  |  |  | *Orgyia antiquoides* Hübner |
|  |  |  | *Orgyia recens* Hübner |
|  |  |  | *Orgyia thyellina* Butler |
|  |  | Noctuidae | *Tiliacea citrago* Linnaeus |
| *Nilea hortulana* (Meigen, 1824) | Lepidoptera | Arctiidae | *Chionarctia nivea* Ménétriés |
|  |  |  | *Hyphantria cunea* Drury |
|  |  |  | *Parasemia plantaginis* Linnaeus |
|  |  | Lymantriidae | *Calliteara pudibunda* Linnaeus |
|  |  |  | *Euproctis similis* Fuessly |
|  |  |  | *Gynaephora selenitica* Esper |
|  |  |  | *Orgyia antiqua* Linnaeus |
|  |  | Noctuidae | *Acronicta aceris* Linnaeus |
|  |  |  | *Acronicta alni* Linnaeus |
|  |  |  | *Acronicta auricoma* Denis & Schiffermüller |
|  |  |  | *Acronicta cuspis* Hübner |
|  |  |  | *Acronicta intermedia* Warren |
|  |  |  | *Acronicta leporina* Linnaeus |
|  |  |  | *Acronicta megacephala* Denis & Schiffermüller |
|  |  |  | *Acronicta psi* Linnaeus |
|  |  |  | *Acronicta rumicis* Linnaeus |
|  |  |  | *Acronicta strigosa* Denis & Schiffermüller |
|  |  |  | *Acronicta tridens* Denis & Schiffermüller |
|  |  |  | *Agrotis segetum* Denis & Schiffermüller |
|  |  |  | *Autographa gamma* Linnaeus |
|  |  |  | *Ceramica pisi* Linnaeus |
|  |  |  | *Cucullia artemisiae* Hufnagel |
|  |  |  | *Mamestra brassicae* Linnaeus |
|  |  |  | *Orthosia cerasi* Fabricius |
|  |  |  | *Xestia ashworthii* Doubleday |
|  |  | Notodontidae | *Phalera bucephala* Linnaeus |
| *Xylotachina diluta* (Meigen, 1824) | Lepidoptera | Cossidae | *Cossus cossus* Linnaeus |
| *Bessa parallela* (Meigen, 1824) | Coleoptera | Coccinellidae | *Epilachna pustulosa* Kono |
|  |  | Curculionidae | *Hypera postica* Gyllenhal |
|  |  |  | *Neoglanis salviae* Schrank |
|  | Hymenoptera | Tenthredinidae | *Craesus japonicus* Takeuchi |
|  |  |  | *Nematus turgaiensis* Safjanov |
|  |  |  | *Pristiphora ezomatsuvora* Togashi |
|  |  |  | *Trichiocampus populi* Okamoto |
|  | Lepidoptera | Arctiidae | *Hyphantria cunea* Drury |
|  |  | Bombycidae | *Bombyx mori* Linnaeus |
|  |  | Chimabachidae | *Diurnea fagella* Denis & Schiffermüller |
|  |  | Crambidae | *Anania terrealis* Treitschke |
|  |  | Drepanidae | *Cilix glaucata* Scopoli |
|  |  | Elachistidae | *Agonopterix heracliana* Linnaeus |
|  |  |  | *Ethmia dodecea* Haworth |
|  |  | Gelechiidae | *Dichomeris oceanis* Meyrick |
|  |  | Geometridae | *Abraxas latifasciata* Warren |
|  |  |  | *Aethalura punctulata* Denis & Schiffermüller |
|  |  |  | *Bupalus piniaria* Linnaeus |
|  |  |  | *Cabera pusaria* Linnaeus |
|  |  |  | *Cyclophora porata* Linnaeus |
|  |  |  | *Cyclophora punctaria* Linnaeus |
|  |  |  | *Cystidia truncangulata* Wehrli |
|  |  |  | *Erannis defoliaria* Clerck |
|  |  |  | *Eupithecia assimilata* Doubleday |
|  |  |  | *Minoa murinata* Scopoli |
|  |  |  | *Naxa seriaria* Motschulsky |
|  |  |  | *Operophtera brunnea* Nakajima |
|  |  |  | *Opisthograptis luteolata* Linnaeus |
|  |  |  | *Tristrophis veneris* Butler |
|  |  | Hesperiidae | *Parnara guttata* Bremer & Grey |
|  |  | Lecithoceridae | *Scythropiodes lividula* Meyrick |
|  |  | Limacodidae | *Monema flavescens* Walker |
|  |  | Lycaenidae | *Neozephyrus japonicus* Murray |
|  |  | Lymantriidae | *Calliteara argentata* Butler |
|  |  |  | *Euproctis pseudoconspersa* Strand |
|  |  |  | *Euproctis subflava* Bremer |
|  |  |  | *Leucoma candida* Staudinger |
|  |  |  | *Leucoma salicis* Linnaeus |
|  |  |  | *Lymantria dispar* Linnaeus |
|  |  |  | *Lymantria fumida* Butler |
|  |  |  | *Lymantria monacha* Linnaeus |
|  |  |  | *Parocneria detrita* Esper |
|  |  |  | *Parocneria furva* Leech |
|  |  | Noctuidae | *Acronicta psi* Linnaeus |
|  |  |  | *Hypena rostralis* Linnaeus |
|  |  |  | *Mythimna separata* Walker |
|  |  |  | *Orthosia miniosa* Denis & Schiffermüller |
|  |  |  | *Perynea subrosea* Butler |
|  |  | Nolidae | *Manoba melancholica* Wileman & West |
|  |  |  | *Nola chlamitulalis* Hübner |
|  |  |  | *Nycteola asiatica* Krulikovsky |
|  |  |  | *Nycteola revayana* Scopoli |
|  |  | Notodontidae | *Clostera pigra* Hufnagel |
|  |  |  | *Notodonta ziczac* Linnaeus |
|  |  | Nymphalidae | *Nymphalis antiopa* Linnaeus |
|  |  | Pieridae | *Pieris brassicae* Linnaeus |
|  |  |  | *Pieris rapae* Linnaeus |
|  |  | Plutellidae | *Plutella xylostella* Linnaeus |
|  |  | Psychidae | *Mahasena aurea* Butler |
|  |  | Pyralidae | *Conobathra bifidella* Leech |
|  |  |  | *Cryptoblabes angustipennella* Ragonot |
|  |  |  | *Sciota marmorata* Alphéraky |
|  |  |  | *Lepidogma tamaricalis* Mann |
|  |  | Thyatiridae | *Tethea or* Denis & Schiffermüller |
|  |  | Tortricidae | *Acleris ferrugana* Denis & Schiffermüller |
|  |  |  | *Acleris rhombana* Denis & Schiffermüller |
|  |  |  | *Adoxophyes orana* Fischer von Röslerstamm |
|  |  |  | *Ancylis mitterbacheriana* Denis & Schiffermüller |
|  |  |  | *Apotomis sauciana* Frölich |
|  |  |  | *Archips crataegana* Hübner |
|  |  |  | *Archips fuscocupreanus* Walsingham |
|  |  |  | *Archips podana* Scopoli |
|  |  |  | *Archips rosana* Linnaeus |
|  |  |  | *Argyrotaenia ljungiana* Thunberg |
|  |  |  | *Cacoecimorpha pronubana* Hübner |
|  |  |  | *Choristoneura diversana* Hübner |
|  |  |  | *Choristoneura murinana* Hübner |
|  |  |  | *Epinotia aciculana* Fal’kovich |
|  |  |  | *Epinotia pygmaeana* Hübner |
|  |  |  | *Eudemis profundana* Denis & Schiffermüller |
|  |  |  | *Eupoecilia ambiguella* Hübner |
|  |  |  | *Grapholita molesta* Busck |
|  |  |  | *Homona issikii* Yasuda |
|  |  |  | *Homona magnanima* Djakonov |
|  |  |  | *Lobesia botrana* Denis & Schiffermüller |
|  |  |  | *Orthotaenia undulana* Denis & Schiffermüller |
|  |  |  | *Pandemis cerasana* Hübner |
|  |  |  | *Pandemis corylana* Fabricius |
|  |  |  | *Pandemis dumetana* Treitschke |
|  |  |  | *Pandemis heparana* Denis & Schiffermüller |
|  |  |  | *Ptycholomoides aeriferana* Herrich-Schäffer |
|  |  |  | *Sparganothis pilleriana* Denis & Schiffermüller |
|  |  |  | *Spilonota eremitana* Moriuti |
|  |  |  | *Strepsicrates rhothia* Meyrick |
|  |  |  | *Tortricodes alternella* Denis & Schiffermüller |
|  |  |  | *Tortrix viridana* Linnaeus |
|  |  |  | *Zeiraphera griseana* Hübner |
|  |  |  | *Zeiraphera rufimitrana* Herrich-Schäffer |
|  |  | Yponomeutidae | *Yponomeuta cagnagella* Hübner |
|  |  |  | *Yponomeuta evonymella* Linnaeus |
|  |  |  | *Yponomeuta mahalebella* Guenée |
|  |  |  | *Yponomeuta malinellus* Zeller |
|  |  |  | *Yponomeuta orientalis* Zagulajev |
|  |  |  | *Yponomeuta padella* Linnaeus |
|  |  |  | *Yponomeuta polystictus* Butler |
|  |  |  | *Yponomeuta polystigmellus* Felder & Felder |
|  |  |  | *Yponomeuta rorrella* Hübner |
|  |  |  | *Yponomeuta tokyonella* Matsumura |
|  |  | Ypsolophidae | *Ypsolopha tsugae* Moriuti |
|  |  |  | *Ypsolopha ustella* Clerck |
|  |  | Zygaenidae | *Artona martini* Efetov |
|  |  |  | *Elcysma westwoodii* Vollenhoven |
|  |  |  | *Illiberis pruni* Dyar |
|  |  |  | *Illiberis rotundata* Jordan |
|  |  |  | *Illiberis tenuis* Butler |
|  |  |  | *Pryeria sinica* Moore |
|  |  |  | *Zygaena trifolii* Esper |
| *Exorista hyalipennis* (Baranov, 1932) | Orthoptera | Tettigoniidae | *Homorocoryphus lineosus* Walker |
| *Phorocera obscura* (Fallén, 1810) | Lepidoptera | Geometridae | *Agriopis aurantiaria* Hübner |
|  |  |  | *Agriopis leucophaearia* Denis & Schiffermüller |
|  |  |  | *Agriopis marginaria* Fabricius |
|  |  |  | *Alsophila aescularia* Denis & Schiffermüller |
|  |  |  | *Arichanna melanaria* Linnaeus |
|  |  |  | *Ectropis crepuscularia* Denis & Schiffermüller |
|  |  |  | *Epirrita dilutata* Denis & Schiffermüller |
|  |  |  | *Erannis defoliaria* Clerck |
|  |  |  | *Operophtera brumata* Linnaeus |
|  |  |  | *Phigalia pilosaria* Denis & Schiffermüller |
|  |  | Noctuidae | *Cosmia trapezina* Linnaeus |
|  |  |  | *Orthosia cerasi* Fabricius |
|  |  |  | *Orthosia cruda* Denis & Schiffermüller |
|  |  |  | *Xestia c-nigrum* Linnaeus |
| *Catagonia aberrans* (Rondani, 1859) | Lepidoptera | Thyrididae | *Thyris fenestrella* Scopoli |
| *Ceromasia rubrifrons* (Macquart, 1834) | Lepidoptera | Arctiidae | *Hyphantria cunea* Drury |
|  |  |  | *Tyria jacobaeae* Linnaeus |
|  |  | Geometridae | *Athroolopha pennigeraria* Hübner |
|  |  |  | *Dyscia lentiscaria* Donzel |
|  |  |  | *Erannis jacobsoni* Djakonov |
|  |  | Hesperiidae | *Carcharodus alceae* Esper |
|  |  | Nymphalidae | *Euphydryas aurinia* Rottemburg |
|  |  | Pieridae | *Aporia crataegi* Linnaeus |
|  |  |  | *Pieris brassicae* Linnaeus |
|  |  | Zygaenidae | *Zygaena alluaudi* Oberthür |
|  |  |  | *Zygaena angelicae* Ochsenheimer |
|  |  |  | *Zygaena carniolica* Scopoli |
|  |  |  | *Zygaena ephialtes* Linnaeus |
|  |  |  | *Zygaena erythrus* Hübner |
|  |  |  | *Zygaena filipendulae* Linnaeus |
|  |  |  | *Zygaena lonicerae* Scheven |
|  |  |  | *Zygaena loti* Denis & Schiffermüller |
|  |  |  | *Zygaena occitanica* Villers |
|  |  |  | *Zygaena purpuralis* Brünnich |
|  |  |  | *Zygaena transalpina* Esper |
|  |  |  | *Zygaena trifolii* Esper |
| *Hebia flavipes* Robineau-Desvoidy, 1830 | Lepidoptera | Geometridae | *Colotois pennaria* Linnaeus |
|  |  | Noctuidae | *Orthosia miniosa* Denis & Schiffermüller |
| *Myxexoristops blondeli* (Robineau-Desvoidy, 1830) | Hymenoptera | Pamphiliidae | *Cephalcia lariciphila* Wachtl |
|  |  |  | *Cephalcia tianmua* Maa |
|  |  |  | *Neurotoma saltuum* Linnaeus |
|  |  | Tenthredinidae | *Mesoneura opaca* Fabricius |
|  |  |  | *Pristiphora erichsonii* Hartig |
| *Pales carbonata* Mesnil, 1970 | Lepidoptera | Arctiidae | *Lemyra imparilis* Butler |
|  |  | Crambidae | *Ostrinia furnacalis* Guenée |
|  |  | Noctuidae | *Camptoloma interiorata* Walker |
|  |  | Pyralidae | *Locastra muscosalis* Walker |
|  |  |  | *Orthaga olivacea* Warren |
|  |  |  | *Sacada approximans* Leech |
| *Pales pavida* (Meigen, 1824) | Lepidoptera | Arctiidae | *Arctia caja* Linnaeus |
|  |  |  | *Atlantarctia tigrina* Villers |
|  |  |  | *Hyphantria cunea* Drury |
|  |  |  | *Hyphoraia aulica* Linnaeus |
|  |  |  | *Ocnogyna baetica* Rambur |
|  |  |  | *Ocnogyna parasita* Hübner |
|  |  |  | *Phragmatobia fuliginosa* Linnaeus |
|  |  |  | *Spilosoma lubricipeda* Linnaeus |
|  |  | Bombycidae | *Bombyx mori* Linnaeus |
|  |  | Crambidae | *Loxostege sticticalis* Linnaeus |
|  |  |  | *Ostrinia latipennis* Warren |
|  |  |  | *Palpita vitrealis* Rossi |
|  |  |  | *Udea* sp.1 |
|  |  |  | *Uresiphita prunipennis* Butler |
|  |  | Geometridae | *Abraxas grossulariata* Linnaeus |
|  |  |  | *Abraxas pantaria* Linnaeus |
|  |  |  | *Agriopis aurantiaria* Hübner |
|  |  |  | *Agriopis bajaria* Denis & Schiffermüller |
|  |  |  | *Agriopis leucophaearia* Denis & Schiffermüller |
|  |  |  | *Agriopis marginaria* Fabricius |
|  |  |  | *Alsophila aceraria* Denis & Schiffermüller |
|  |  |  | *Alsophila aescularia* Denis & Schiffermüller |
|  |  |  | *Aplocera efformata* Guenée |
|  |  |  | *Biston strataria* Hufnagel |
|  |  |  | *Chesias linogrisearia* Constant |
|  |  |  | *Coenotephria salicata* Denis & Schiffermüller |
|  |  |  | *Coenotephria tophaceata* Denis & Schiffermüller |
|  |  |  | *Crocallis tusciaria* Borkhausen |
|  |  |  | *Cystidia couaggaria* Guenée |
|  |  |  | *Cystidia truncangulata* Wehrli |
|  |  |  | *Ennomos autumnaria* Werneburg |
|  |  |  | *Ennomos quercinaria* Hufnagel |
|  |  |  | *Erannis defoliaria* Clerck |
|  |  |  | *Eupithecia assimilata* Doubleday |
|  |  |  | *Hypomecis punctinalis* Scopoli |
|  |  |  | *Lycia lapponaria* Boisduval |
|  |  |  | *Odontopera bidentata* Clerck |
|  |  |  | *Pasiphila excisa* Butler |
|  |  |  | *Pelurga comitata* Linnaeus |
|  |  |  | *Perizoma alchemillata* Linnaeus |
|  |  |  | *Xanthorrhoe montanata* Denis & Schiffermüller |
|  |  | Hesperiidae | *Isoteinon lamprospilus* Felder & Felder |
|  |  |  | *Thymelicus acteon* Rottemburg |
|  |  |  | *Thymelicus lineola* Ochsenheimer |
|  |  | Lasiocampidae | *Cosmotriche lobulina* Denis & Schiffermüller |
|  |  |  | *Dendrolimus pini* Linnaeus |
|  |  |  | *Dendrolimus superans* Butler |
|  |  |  | *Eriogaster catax* Linnaeus |
|  |  |  | *Eriogaster lanestris* Linnaeus |
|  |  |  | *Lasiocampa quercus* Linnaeus |
|  |  |  | *Lasiocampa trifolii* Denis & Schiffermüller |
|  |  |  | *Malacosoma castrensis* Linnaeus |
|  |  |  | *Malacosoma franconica* Denis & Schiffermüller |
|  |  |  | *Malacosoma neustria* Linnaeus |
|  |  |  | *Malacosoma parallela* Staudinger |
|  |  | Libytheidae | *Libythea celtis* Laicharting |
|  |  | Limacodidae | *Parasa consocia* Walker |
|  |  |  | *Parasa sinica* Moore |
|  |  |  | *Phrixolepia sericea* Butler |
|  |  | Lycaenidae | *Favonius orientalis* Murray |
|  |  |  | *Favonius ultramarinus* Fixsen |
|  |  |  | *Favonius yuasai* Shirôz |
|  |  |  | *Lycaena dispar* Haworth |
|  |  |  | *Lycaena phlaeas* Linnaeus |
|  |  | Lymantriidae | *Calliteara pudibunda* Linnaeus |
|  |  |  | *Euproctis chrysorrhoea* Linnaeus |
|  |  |  | *Euproctis kargalica* Moore |
|  |  |  | *Euproctis pseudoconspersa* Strand |
|  |  |  | *Euproctis similis* Fuessly |
|  |  |  | *Gynaephora selenitica* Esper |
|  |  |  | *Ivela auripes* Butler |
|  |  |  | *Laelia coenosa* Hübner |
|  |  |  | *Leucoma salicis* Linnaeus |
|  |  |  | *Lymantria dispar* Linnaeus |
|  |  |  | *Lymantria monacha* Li |
|  |  |  | *Orgyia antiqua* Linnaeus |
|  |  |  | *Orgyia antiquoides* Hübner |
|  |  |  | *Orgyia dubia* Tauscher |
|  |  |  | *Penthophera morio* Linnaeus |
|  |  | Noctuidae | *Acosmetia caliginosa* Hübner |
|  |  |  | *Acronicta aceris* Linnaeus |
|  |  |  | *Acronicta alni* Linnaeus |
|  |  |  | *Acronicta major* Bremer |
|  |  |  | *Acronicta megacephala* Denis & Schiffermüller |
|  |  |  | *Acronicta psi* Linnaeus |
|  |  |  | *Acronicta rumicis* Linnaeus |
|  |  |  | *Acronicta tridens* Denis & Schiffermüller |
|  |  |  | *Agrochola litura* Linnaeus |
|  |  |  | *Agrotis bigramma* Esper |
|  |  |  | *Agrotis exclamationis* Linnaeus |
|  |  |  | *Agrotis segetum* Denis & Schiffermüller |
|  |  |  | *Agrotis trux* Hübner or *Noctua comes* Hübner |
|  |  |  | *Allophyes oxyacanthae* Linnaeus |
|  |  |  | *Amphipyra livida* Denis & Schiffermüller |
|  |  |  | *Amphipyra pyramidea* Linnaeus |
|  |  |  | *Anorthoa munda* Denis & Schiffermüller |
|  |  |  | *Antitype chi* Linnaeus |
|  |  |  | *Apamea anceps* Denis & Schiffermüller |
|  |  |  | *Apamea crenata* Hufnagel |
|  |  |  | *Apamea unanimis* Hübner |
|  |  |  | *Apopestes spectrum* Esper |
|  |  |  | *Athetis hospes* Freyer |
|  |  |  | *Autographa gamma* Linnaeus |
|  |  |  | *Axylia putris* Linnaeus |
|  |  |  | *Calophasia lunula* Hufnagel |
|  |  |  | *Camptoloma interiorata* Walker |
|  |  |  | *Caradrina* sp.1 |
|  |  |  | *Catocala nymphagoga* Esper |
|  |  |  | *Ceramica pisi* Linnaeus |
|  |  |  | *Cerapteryx graminis* Linnaeus |
|  |  |  | *Chilodes maritima* Tauscher |
|  |  |  | *Chloantha hyperici* Denis & Schiffermüller |
|  |  |  | *Chrysodeixis chalcites* Esper |
|  |  |  | *Cosmia trapezina* Linnaeus |
|  |  |  | *Cucullia asteris* Denis & Schiffermüller |
|  |  |  | *Cucullia prenanthis* Boisduval |
|  |  |  | *Cucullia umbratica* Linnaeus |
|  |  |  | *Cucullia verbasci* Linnaeus |
|  |  |  | *Diarsia brunnea* Denis & Schiffermüller |
|  |  |  | *Diloba caeruleocephala* Linnaeus |
|  |  |  | *Dryobotodes eremita* Fabricius |
|  |  |  | *Dryobotodes monochroma* Esper |
|  |  |  | *Euplexia lucipara* Linnaeus |
|  |  |  | *Helicoverpa armigera* Hübner |
|  |  |  | *Helicoverpa assulta* Guenée |
|  |  |  | *Heliothis viriplaca* Hufnagel |
|  |  |  | *Leucania loreyi* Duponchel |
|  |  |  | *Lacanobia blenna* Hübner |
|  |  |  | *Lacanobia oleracea* Linnaeus |
|  |  |  | *Lithophane ornitopus* Hufnagel |
|  |  |  | *Macdunnoughia confusa* Stephens |
|  |  |  | *Mamestra brassicae* Linnaeus |
|  |  |  | *Moma alpium* Osbeck |
|  |  |  | *Mythimna ferrago* Fabricius |
|  |  |  | *Mythimna impura* Hübner |
|  |  |  | *Mythimna pallens* Linnaeus |
|  |  |  | *Mythimna separata* Walker |
|  |  |  | *Mythimna straminea* Treitschke |
|  |  |  | *Mythimna unipuncta* Haworth |
|  |  |  | *Naenia typica* Linnaeus |
|  |  |  | *Noctua pronuba* Linnaeus |
|  |  |  | *Ochropleura leucogaster* Freyer |
|  |  |  | *Orthosia carnipennis* Butler |
|  |  |  | *Orthosia cerasi* Fabricius |
|  |  |  | *Orthosia cruda* Denis & Schiffermüller |
|  |  |  | *Orthosia gothica* Linnaeus |
|  |  |  | *Orthosia gracilis* Denis & Schiffermüller |
|  |  |  | *Orthosia incerta* Hufnagel |
|  |  |  | *Orthosia miniosa* Denis & Schiffermüller |
|  |  |  | *Panolis flammea* Denis & Schiffermüller |
|  |  |  | *Peridroma saucia* Hübner |
|  |  |  | *Phlogophora meticulosa* Linnaeus |
|  |  |  | *Simyra albovenosa* Goeze |
|  |  |  | *Simyra dentinosa* Freyer |
|  |  |  | *Spodoptera cilium* Guenée |
|  |  |  | *Spodoptera exigua* Hübner |
|  |  |  | *Spodoptera litura* Fabricius |
|  |  |  | *Tiliacea citrago* Linnaeus |
|  |  |  | *Tyta luctuosa* Denis & Schiffermüller |
|  |  |  | *Xestia stigmatica* Hübner |
|  |  |  | *Xestia xanthographa* Denis & Schiffermüller |
|  |  |  | *Xylena formosa* Butler |
|  |  |  | *Xylena solidaginis* Hübner |
|  |  |  | *Xylena vetusta* Hübner |
|  |  | Nolidae | *Iscadia uniformis* Inoue & Sugi |
|  |  |  | *Nola* sp.1 |
|  |  |  | *Nycteola siculana* Fuchs |
|  |  |  | *Pseudoips prasinana* Linnaeus |
|  |  | Notodontidae | *Cerura vinula* Linnaeus |
|  |  |  | *Drymonia* sp.1 |
|  |  |  | *Peridea anceps* Goeze |
|  |  |  | *Phalera bucephala* Linnaeus |
|  |  |  | *Phalera bucephaloides* Ochsenheimer |
|  |  |  | *Ptilodon capucina* Linnaeus |
|  |  |  | *Syntypistis punctatella* Motschulsky |
|  |  | Nymphalidae | *Aglais ichnusa* Bonelli |
|  |  |  | *Aglais urticae* Linnaeus |
|  |  |  | *Argynnis paphia* Linnaeus |
|  |  |  | *Boloria selene* Denis & Schiffermüller |
|  |  |  | *Inachis io* Linnaeus |
|  |  |  | *Melitaea ambigua* Ménétriés |
|  |  |  | *Melitaea athalia* Rottemburg |
|  |  |  | *Neptis philyra* Ménétriés |
|  |  |  | *Nymphalis xanthomelas* Esper |
|  |  |  | *Vanessa atalanta* Linnaeus |
|  |  |  | *Vanessa indica* Herbst |
|  |  | Papilionidae | *Parnassius apollo* Linnaeus |
|  |  | Pieridae | *Aporia crataegi* Linnaeus |
|  |  |  | *Colias croceus* Fourcroy |
|  |  |  | *Pieris brassicae* Linnaeus |
|  |  | Psychidae | *Eumeta minuscula* Butler |
|  |  |  | *Sterrhopterix standfussi* Wocke |
|  |  | Pterophoridae | *Cnaemidophorus rhododactyla* Denis & Schiffermüller |
|  |  |  | *Oidaematophorus lithodactyla* Treitschke |
|  |  | Pyralidae | *Conobathra bifidella* Leech |
|  |  |  | *Phycita roborella* Denis & Schiffermüller |
|  |  | Saturniidae | *Samia cynthia* Drury |
|  |  |  | *Saturnia japonica* Moore |
|  |  | Satyridae | *Coenonympha tullia* Müller |
|  |  |  | *Hipparchia semele* Linnaeus |
|  |  |  | *Pararge aegeria* Linnaeus |
|  |  | Sphingidae | *Deilephila elpenor* Linnaeus |
|  |  |  | *Hyles euphorbiae* Linnaeus |
|  |  |  | *Laothoe populi* Linnaeus |
|  |  |  | *Mimas tiliae* Linnaeus |
|  |  | Thaumetopoeidae | *Thaumetopoea pityocampa* Denis & Schiffermüller |
|  |  |  | *Thaumetopoea processionea* Linnaeus |
|  |  |  | *Thaumetopoea solitaria* Freyer |
|  |  | Thyatiridae | *Achlya flavicornis* Linnaeus |
|  |  |  | *Asphalia ruficollis* Denis & Schiffermüller |
|  |  |  | *Polyploca ridens* Fabricius |
|  |  | Tortricidae | *Acleris ferrugana* Denis & Schiffermüller |
|  |  |  | *Acleris hastiana* Linnaeus |
|  |  |  | *Aphelia viburniana* Denis & Schiffermüller |
|  |  |  | *Archips fuscocupreanus* Walsingham |
|  |  |  | *Cacoecimorpha pronubana* Hübner |
|  |  |  | *Gypsonoma nitidulana* Lienig & Zeller |
|  |  |  | *Pandemis heparana* Denis & Schiffermüller |
|  |  |  | *Ptycholomoides aeriferana* Herrich-Schäffer |
|  |  |  | *Sparganothis pilleriana* Denis & Schiffermüller |
|  |  |  | *Tortrix viridana* Linnaeus |
|  |  |  | *Zeiraphera griseana* Hübner |
|  |  | Yponomeutidae | *Yponomeuta evonymella* Linnaeus |
|  |  | Zygaenidae | *Elcysma westwoodii* Vollenhoven |
|  |  |  | *Illiberis pruni* Dyar |
|  |  |  | *Illiberis rotundata* Jordan |
|  |  |  | *Pidorus atratus* Butler |
|  |  |  | *Zygaena carniolica* Scopoli |
|  |  |  | *Zygaena filipendulae* Linnaeus |
|  |  |  | *Zygaena lonicerae* Scheven |
|  |  |  | *Zygaena trifolii* Esper |
| *Phryno vetula* (Meigen, 1824) | Lepidoptera | Geometridae | *Agriopis aurantiaria* Hübner |
|  |  |  | *Agriopis bajaria* Denis & Schiffermüller |
|  |  |  | *Erannis defoliaria* Clerck |
|  |  |  | *Hydriomena ruberata* Freyer |
|  |  |  | *Lycia hirtaria* Clerck |
|  |  |  | *Operophtera brumata* Linnaeus |
|  |  |  | *Phigalia pilosaria* Denis & Schiffermüller |
|  |  | Lasiocampidae | *Lasiocampa quercus* Linnaeus |
|  |  | Noctuidae | *Anorthoa munda* Denis & Schiffermüller |
|  |  |  | *Cosmia trapezina* Linnaeus |
|  |  |  | *Cucullia prenanthis* Boisduval |
|  |  |  | *Dryobotodes eremita* Fabricius |
|  |  |  | *Griposia aprilina* Linnaeus |
|  |  |  | *Orthosia cerasi* Fabricius |
|  |  |  | *Orthosia miniosa* Denis & Schiffermüller |
|  |  | Thyatiridae | *Cymatophorima diluta* Denis & Schiffermüller |
| *Scaphimyia takanoi* Mesnil, 1967 | Lepidoptera | Hepialidae | *Endoclita excrescens* Butler |
| *Winthemia quadripustulata* (Fabricius, 1794) | Lepidoptera | Arctiidae | *Arctia caja* Linnaeus |
|  |  |  | *Phragmatobia fuliginosa* Linnaeus |
|  |  |  | *Tyria jacobaeae* Linnaeus |
|  |  | Geometridae | *Ectropis crepuscularia* Denis & Schiffermüller |
|  |  |  | *Larentia clavaria* Haworth |
|  |  |  | *Lycia hirtaria* Clerck |
|  |  |  | *Lycia zonaria* Denis & Schiffermüller |
|  |  |  | *Ourapteryx sambucaria* Linnaeus |
|  |  | Lasiocampidae | *Malacosoma neustria* Linnaeus |
|  |  | Lymantriidae | *Leucoma salicis* Linnaeus |
|  |  |  | *Lymantria dispar* Linnaeus |
|  |  | Noctuidae | *Acronicta tridens* Denis & Schiffermüller |
|  |  |  | *Agrotis* sp.1 |
|  |  |  | *Ammoconia caecimacula* Denis & Schiffermüller |
|  |  |  | *Autographa gamma* Linnaeus |
|  |  |  | *Calophasia lunula* Hufnagel |
|  |  |  | *Ceramica pisi* Linnaeus |
|  |  |  | *Cerapteryx graminis* Linnaeus |
|  |  |  | *Cucullia argentea* Hufnagel |
|  |  |  | *Cucullia artemisiae* Hufnagel |
|  |  |  | *Cucullia asteris* Denis & Schiffermüller |
|  |  |  | *Cucullia blattariae* Esper |
|  |  |  | *Cucullia lactucae* Denis & Schiffermüller |
|  |  |  | *Cucullia lanceolata* Villers |
|  |  |  | *Cucullia lucifuga* Denis & Schiffermüller |
|  |  |  | *Cucullia lychnitis* Rambur |
|  |  |  | *Cucullia prenanthis* Boisduval |
|  |  |  | *Cucullia scopariae* Dorfmeister |
|  |  |  | *Cucullia scrophulariae* Denis & Schiffermüller |
|  |  |  | *Cucullia umbratica* Linnaeus |
|  |  |  | *Cucullia verbasci* Linnaeus |
|  |  |  | *Diloba caeruleocephala* Linnaeus |
|  |  |  | *Euchalcia variabilis* Piller |
|  |  |  | *Gortyna flavago* Denis & Schiffermüller |
|  |  |  | *Hadena bicruris* Hufnagel |
|  |  |  | *Helicoverpa armigera* Hübner |
|  |  |  | *Heliothis peltigera* Denis & Schiffermüller |
|  |  |  | *Lacanobia oleracea* Linnaeus |
|  |  |  | *Lenisa geminipuncta* Haworth |
|  |  |  | *Mamestra brassicae* Linnaeus |
|  |  |  | *Mythimna unipuncta* Haworth |
|  |  |  | *Orthosia cruda* Denis & Schiffermüller |
|  |  |  | *Phlogophora meticulosa* Linnaeus |
|  |  |  | *Polychrysia moneta* Fabricius |
|  |  |  | *Xestia c-nigrum* Linnaeus |
|  |  | Notodontidae | *Cerura vinula* Linnaeus |
|  |  | Nymphalidae | *Aglais urticae* Linnaeus |
|  |  |  | *Argynnis aglaja* Linnaeus |
|  |  |  | *Inachis io* Linnaeus |
|  |  | Papilionidae | *Zerynthia polyxena* Denis & Schiffermüller |
|  |  | Sphingidae | *Deilephila elpenor* Linnaeus |
|  |  |  | *Deilephila porcellus* Linnaeus |
|  |  |  | *Hyles euphorbiae* Linnaeus |
|  |  |  | *Laothoe populi* Linnaeus |
|  |  |  | *Smerinthus ocellata* Linnaeus |
|  |  | Tortricidae | *Aphelia paleana* Hübner |
| *Winthemia venusta* (Meigen, 1824) | Lepidoptera | Geometridae | *Ascotis selenaria* Denis & Schiffermüller |
|  |  |  | *Lycia hirtaria* Clerck |
|  |  | Lasiocampidae | *Dendrolimus superans* Butler |
|  |  | Lymantriidae | *Lymantria dispar* Linnaeus |
|  |  | Noctuidae | *Catocala sponsa* Linnaeus |
|  |  |  | *Eudocima salaminia* Cramer |
|  |  |  | *Eudocima tyrannus* Guenée |
|  |  |  | *Scoliopteryx libatrix* Linnaeus |
|  |  | Nymphalidae | *Nymphalidae* sp.1 |
|  |  | Notodontidae | *Stauropus fagi* Linnaeus |
|  |  |  | *Torigea straminea* Moore |
|  |  | Thaumetopoeidae | *Thaumetopoea processionea* Linnaeus |
| *Ectophasia crassipennis* (Fabricius, 1794) | Heteroptera | Acanthosomatidae | *Acanthosoma haemorrhoidale* Linnaeus |
|  |  |  | *Elasmostethus* sp.1 |
|  |  |  | *Elasmucha grisea* Linnaeus |
|  |  | Coreidae | *Coreus marginatus* Linnaeus |
|  |  |  | *Gonocerus acuteangulatus* Goeze |
|  |  | Lygaeidae | *Spilostethus saxatilis* Scopoli |
|  |  |  | *Tropidothorax leucopterus* Goeze |
|  |  | Pentatomidae | *Aelia acuminata* Linnaeus |
|  |  |  | *Aelia furcula* Fieber |
|  |  |  | *Aelia rostrata* Boheman |
|  |  |  | *Arma custos* Fabricius |
|  |  |  | *Carpocoris fuscispinus* Boheman |
|  |  |  | *Carpocoris pudicus* Poda |
|  |  |  | *Carpocoris purpureipennis* De Geer |
|  |  |  | *Dolycoris baccarum* Linnaeus |
|  |  |  | *Eurydema oleracea* Linnaeus |
|  |  |  | *Eurydema ornata* Linnaeus |
|  |  |  | *Eurydema rugosa* Motschulsky |
|  |  |  | *Eurydema ventralis* Kolenati |
|  |  |  | *Graphosoma lineatum* Linnaeus |
|  |  |  | *Graphosoma rubrolineatum* Westwood |
|  |  |  | *Menida disjecta* Uhler |
|  |  |  | *Nezara viridula* Linnaeus |
|  |  |  | *Palomena angulosa* Motschulsky |
|  |  |  | *Palomena prasina* Linnaeus |
|  |  |  | *Peribalus strictus* Fabricius |
|  |  |  | *Piezodorus lituratus* Fabricius |
|  |  |  | *Rhaphigaster nebulosa* Poda |
|  |  | Reduviidae | *Rhynocoris annulatus* Linnaeus |
|  |  |  | *Rhynocoris leucospilus* Stål |
|  |  | Scutelleridae | *Eurygaster austriaca* Schrank |
|  |  |  | *Eurygaster hottentotta* Fabricius |
|  |  |  | *Eurygaster integriceps* Puton |
|  |  |  | *Eurygaster maura* Linnaeus |
|  |  |  | *Odontotarsus purpureolineatus* Rossi |
| *Ectophasia rotundiventris* (Loew, 1858) | Heteroptera | Acanthosomatidae | *Acanthosoma denticaudum* Jakovlev |
|  |  |  | *Acanthosoma haemorrhoidale* Linnaeus |
|  |  |  | *Acanthosoma labiduroides* Jakovlev |
|  |  | Pentatomidae | *Carbula humerigera* Uhler |
|  |  |  | *Dolycoris baccarum* Linnaeus |
|  |  |  | *Eurydema rugosa* Motschulsky |
|  |  |  | *Eysarcoris lewisi* Distant |
|  |  |  | *Glaucias subpunctatus* Walker |
|  |  |  | *Gonopsis affinis* Uhler |
|  |  |  | *Lelia decempunctata* Motschulsky |
|  |  |  | *Menida disjecta* Uhler |
|  |  |  | *Menida musiva*  Jakovlev |
|  |  |  | *Palomena angulosa* Motschulsky |
|  |  |  | *Palomena viridissima* Poda |
|  |  |  | *Pentatoma japonica* Distant |
|  |  |  | *Plautia crossota* Dallas |
| *Subclytia rotundiventris* (Fallén, 1820) | Heteroptera | Acanthosomatidae | *Cyphostethus tristriatus* Fabricius |
|  |  |  | *Elasmostethus interstinctus* Linnaeus |
|  |  |  | *Elasmucha betulae* De Geer |
|  |  |  | *Elasmucha grisea* Linnaeus |
|  |  | Pentatomidae | *Aelia acuminata* Linnaeus |
|  |  |  | *Piezodorus lituratus* Fabricius |
| *Linnaemya tessellans* (Robineau-Desvoidy, 1830) | Lepidoptera | Noctuidae | *Xestia c*-*nigrum* Linnaeus |
| *Phytomyptera zonella* (Zetterstedt, 1844) | Lepidoptera | Tortricidae | *Epinotia aciculana* Fal’kovich |
| *Bithia demotica* (Egger, 1861) | Lepidoptera | Sesiidae | *Bembecia dispar* Staudinger |
|  |  |  | *Bembecia ichneumoniformis* Denis & Schiffermüller |
|  |  |  | *Bembecia lomatiaeformis* Lederer |
|  |  |  | *Bembecia pavicevici* Toševski |
|  |  |  | *Bembecia psoraleae* Bartsch & Bettag |
|  |  |  | *Bembecia sanguinolenta* Lederer |
|  |  |  | *Bembecia scopigera* Scopoli |
|  |  |  | *Bembecia sirphiformis* Lucas |
|  |  |  | *Chamaesphecia masariformis* Ochsenheimer |
|  |  |  | *Chamaesphecia proximata* Staudinger |
|  |  |  | *Pyropteron affinis* Staudinger |
|  |  |  | *Pyropteron doryliformis* Ochsenheimer |
|  |  |  | *Pyropteron triannuliformis* Freyer |
| *Bithia modesta* (Meigen, 1824) | Lepidoptera | Sesiidae | *Bembecia apyra* Le Cerf |
|  |  |  | *Bembecia megillaeformis* Hübner |
|  |  |  | *Bembecia pontica* Staudinger |
|  |  |  | *Bembecia scopigera* Scopoli |
|  |  |  | *Bembecia sirphiformis* Lucas |
|  |  |  | *Bembecia stiziformis* Herrich-Schäffer |
|  |  |  | *Chamaesphecia crassicornis* Bartel |
|  |  |  | *Pyropteron affinis* Staudinger |
|  |  |  | *Pyropteron chrysidiformis* Esper |
|  |  |  | *Pyropteron doryliformis* Ochsenheimer |
|  |  |  | *Pyropteron hispanica* Kallies |
|  |  |  | *Pyropteron koschwitzi* Špatenka |
|  |  |  | *Pyropteron leucomelaena* Zeller |
|  |  |  | *Pyropteron meriaeformis* Boisduval |
|  |  |  | *Pyropteron triannuliformis* Freyer |
| *Linnaemya atriventris* (Malloch, 1935) | Lepidoptera | Noctuidae | *Pseudalelimma miwai* Inoue |
| *Linnaemya omega* Zimin, 1954 | Lepidoptera | Lymantriidae | *Leucoma salicis* Linnaeus |
| *Linnaemya picta* (Meigen, 1824) | Lepidoptera | Noctuidae | *Agrotis* sp.1 |
|  |  |  | *Anaplectoides prasina* Denis & Schiffermüller |
|  |  |  | *Diarsia brunnea* Denis & Schiffermüller |
|  |  |  | *Eugraphe sigma* Denis & Schiffermüller |
|  |  |  | *Graphiphora augur* Fabricius |
|  |  |  | *Mamestra brassicae* Linnaeus |
|  |  |  | *Naenia typica* Linnaeus |
|  |  |  | *Noctua comes* Hübner |
|  |  |  | *Xestia c*-*nigrum* Linnaeus |
|  |  |  | *Xestia triangulum* Hufnagel |
| *Macquartia tessellum* (Meigen, 1824) | Coleoptera | Chrysomelidae | *Chrysolina americana* Linnaeus |
|  |  |  | *Chrysolina didymata* Scriba |
|  |  |  | *Chrysolina geminata* Paykull |
|  |  |  | *Chrysolina hyperici* Forster |
|  |  |  | *Chrysolina varians* Schaller |
|  |  |  | *Colaphus palaestinus* Achard |
|  |  |  | *Colaphus sophiae* Schaller |
|  |  |  | *Entomoscelis adonidis* Pallas |
|  |  |  | *Gonioctena olivacea* Forster |
| *Actia crassicornis* (Meigen, 1824) | Lepidoptera | Elachistidae | *Agonopterix alpigena* Frey |
|  |  |  | *Agonopterix angelicella* Hübner |
|  |  |  | *Agonopterix astrantiae* HeineMann |
|  |  |  | *Agonopterix cnicella* Treitschke |
|  |  |  | *Agonopterix conterminella* Zeller |
|  |  |  | *Agonopterix heracliana* Linnaeus |
|  |  |  | *Agonopterix hippomarathri* Nickerl |
|  |  |  | *Agonopterix liturosa* Haworth |
|  |  |  | *Agonopterix nervosa* Haworth |
|  |  |  | *Agonopterix purpurea* Haworth |
|  |  |  | *Agonopterix scopariella* HeineMann |
|  |  |  | *Agonopterix subpropinquella* Stainton |
|  |  |  | *Agonopterix umbellana* Fabricius |
|  |  |  | *Depressaria bupleurella* HeineMann |
|  |  |  | *Depressaria depressana* Fabricius |
|  |  |  | *Depressaria heydenii* Zeller |
|  |  |  | *Depressaria marcella* Rebel |
|  |  | Gelechiidae | *Mirificarma lentiginosella* Zeller |
|  |  | Plutellidae | *Rhigognostis incarnatella* Steudel |
|  |  | Tortricidae | *Acleris* sp.1 |
|  |  |  | *Ancylis mitterbacheriana* Denis & Schiffermüller |
|  |  |  | *Sparganothis pilleriana* Denis & Schiffermüller |
|  |  |  | *Tortrix viridana* Linnaeus |
|  |  |  | *Zeiraphera isertana* Fabricius |
| *Peribaea abbreviata* Tachi & Shima, 2002 | Lepidoptera | Geometridae | *Endropiodes circumflexus* Inoue |
| *Peribaea glabra* Tachi & Shima, 2002 | Lepidoptera | Drepanidae | *Oreta turpis* Butler |
| *Peribaea tibialis* (Robineau-Desvoidy, 1851) | Lepidoptera | Arctiidae | *Eilema complana* Linnaeus |
|  |  |  | *Phragmatobia fuliginosa* Linnaeus |
|  |  | Crambidae | *Ostrinia nubilalis* Hübner |
|  |  | Geometridae | *Alcis repandata* Linnaeus |
|  |  |  | *Angerona prunaria* Linnaeus |
|  |  |  | *Biston betularia* Linnaeus |
|  |  |  | *Cepphis advenaria* Hübner |
|  |  |  | *Cleora cinctaria* Denis & Schiffermüller |
|  |  |  | *Cyclophora puppillaria* Hübner |
|  |  |  | *Dysstroma truncata* Hufnagel |
|  |  |  | *Ectropis crepuscularia* Denis & Schiffermüller |
|  |  |  | *Ematurga atomaria* Linnaeus |
|  |  |  | *Ennomos alniaria* Linnaeus |
|  |  |  | *Ennomos autumnaria* Werneburg |
|  |  |  | *Ennomos quercinaria* Hufnagel |
|  |  |  | *Ennomos erosaria* Denis & Schiffermüller |
|  |  |  | *Eulithis populata* Linnaeus |
|  |  |  | *Lycia hirtaria* Clerck |
|  |  |  | *Macaria notata* Linnaeus |
|  |  |  | *Pachycnemia hippocastanaria* Hübner |
|  |  |  | *Scopula nigropunctata* Hufnagel |
|  |  |  | *Scopula ternata* Schrank |
|  |  | Lasiocampidae | *Lasiocampa grandis* Rogenhofer |
|  |  |  | *Lasiocampa terreni* Herrich-Schäffer |
|  |  | Lymantriidae | *Lymantria dispar* Linnaeus |
|  |  |  | *Orgyia dubia* Tauscher |
|  |  | Noctuidae | *Ammoconia caecimacula* Denis & Schiffermüller |
|  |  |  | *Anarta myrtilli* Linnaeus |
|  |  |  | *Aporophyla lutulenta* Denis & Schiffermüller |
|  |  |  | *Cucullia formosa* Rogenhofer |
|  |  |  | *Euclidia mi* Clerck |
|  |  |  | *Lacanobia oleracea* Linnaeus |
|  |  |  | *Lygephila pastinum* Treitschke |
|  |  |  | *Mamestra brassicae* Linnaeus |
|  |  |  | *Mythimna unipuncta* Haworth |
|  |  |  | *Noctua interjecta* Hübner |
|  |  |  | *Orthosia cerasi* Fabricius |
|  |  |  | *Orthosia miniosa* Denis & Schiffermüller |
|  |  |  | *Phyllophila obliterata* Rambur |
|  |  |  | *Polymixis flavicincta* Denis & Schiffermüller |
|  |  |  | *Pyrrhia umbra* Hufnagel |
|  |  |  | *Sesamia nonagrioides* Lefebvre |
|  |  |  | *Spodoptera exigua* Hübner |
|  |  |  | *Tarachidia candefacta* Hübner |
|  |  | Notodontidae | *Furcula furcula* Clerck |
|  |  | Satyridae | Satyridae sp.1 |
